# Supplementary figures and images for: Inferring latent temporal progression and regulatory networks from cross-sectional transcriptomic data of cancer samples
Source: PLoS Comput Biol. 2021 Mar 5;17(3):e1008379. doi: 10.1371/journal.pcbi.1008379 (PMC7968745; doi:10.1371/journal.pcbi.1008379)

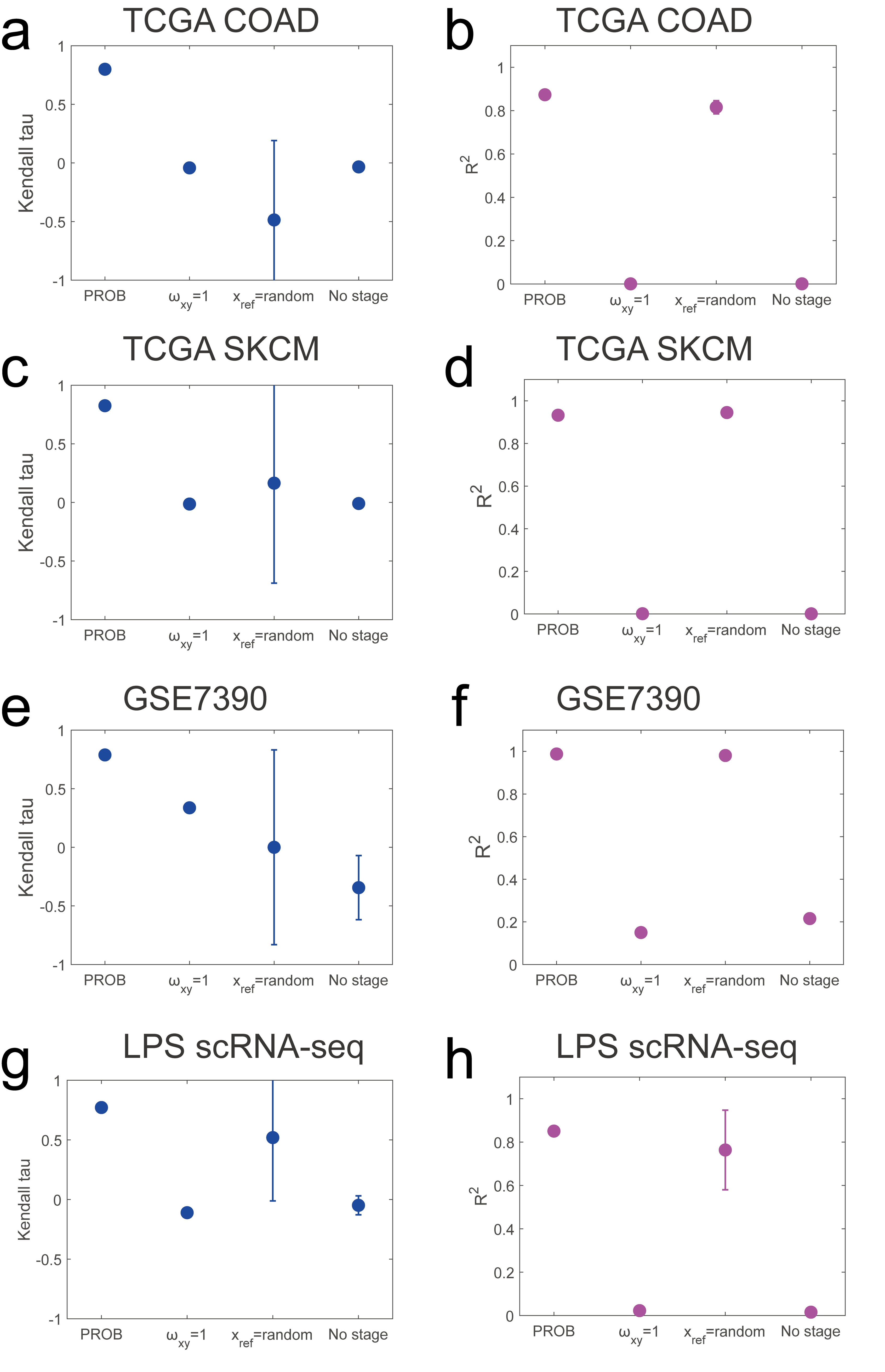

Supplement: S1 Fig — We compared PROB with its several variants: ‘ωxy = 1’ represents setting the weight coefficient ωxy in Eq (2) to be 1; ‘xref = random’ represents randomly assigning the reference point (Eq (9)) to identify the rooting point as the previous pseudotime inference methods usually did; ‘No stage’ represents leaving out the stage information, i.e., both ‘ωxy = 1’ and ‘xref = random’. Kendall tau correlation coefficient or determinant coefficient (R2) between the inferred temporal progression and the staging data (or the capture time in scRNA-seq data) was calculated for each method. (a) Kendall tau for the TCGA COAD dataset. (b) R2 for the TCGA COAD dataset. (c) Kendall tau for the TCGA SKCM dataset. (d) R2 for the TCGA SKCM dataset. (e) Kendall tau for the GSE7390 dataset. (f) R2 for the GSE7390 dataset. (g) Kendall tau for the LPS scRNA-seq dataset. (h) R2 for the LPS scRNA-seq dataset. (TIF) [file pcbi.1008379.s001.tif]

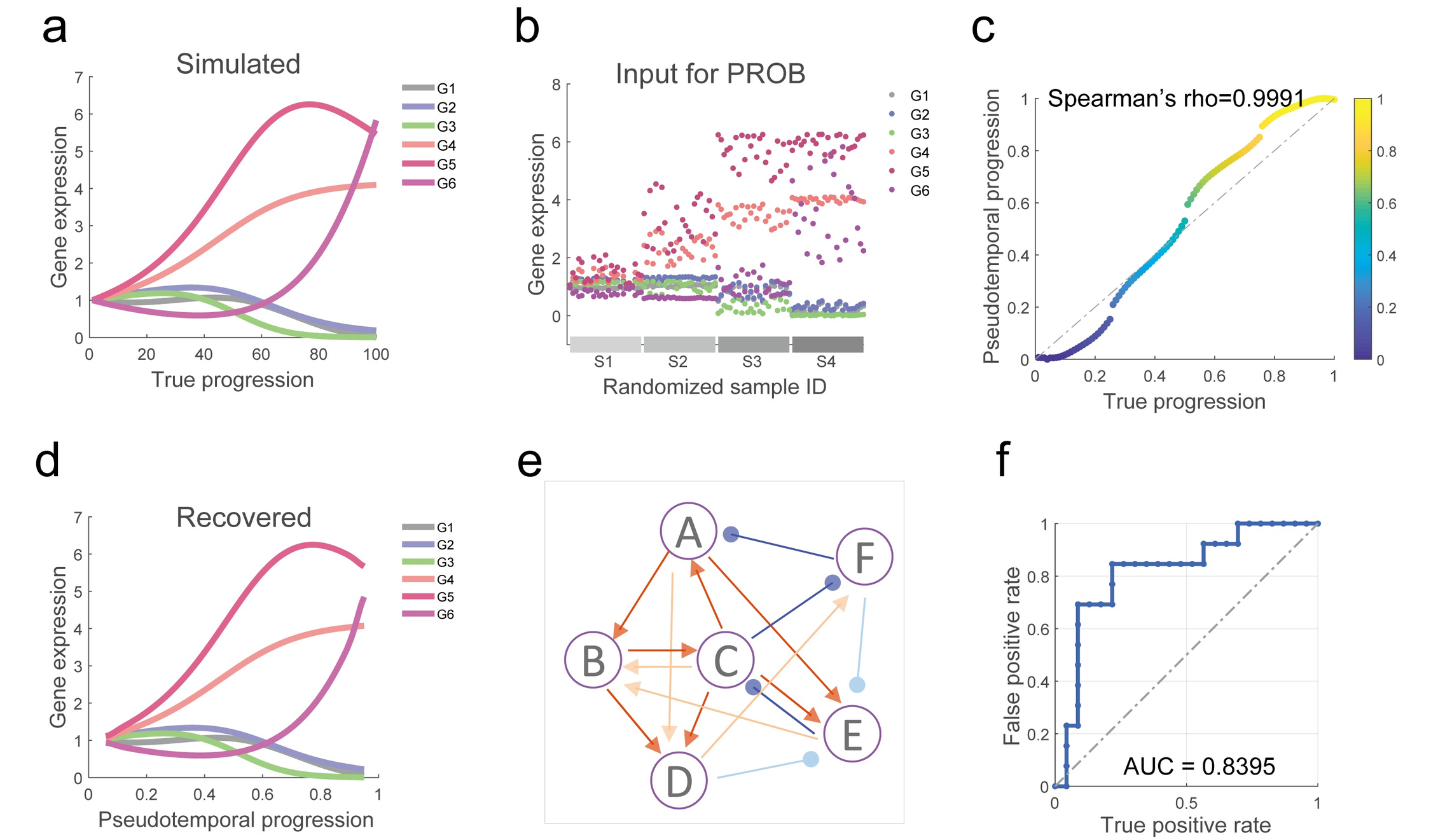

Supplement: S2 Fig — (a) A set of synthetic gene expression data of 100 cancer patients along with true progression. For illustration and visualization purpose, only 6 genes were tested. (b) Simulated tumor sample-based gene expression data by randomizing sample IDs of data in (a) but retaining staging information, which was used as input for PROB. (c) Comparison of inferred temporal progression with true progression in the synthetic dataset. (d) Recovered gene expression dynamics along with temporal progression. (e) The inferred GRN using Bayesian LASSO method based on data in (d). (f) Accuracy of GRN inference evaluated using area under curve (AUC) of ROC for the inferred network compared to the ground-truth network (AUC = 0.8395). (TIF) [file pcbi.1008379.s002.tif]

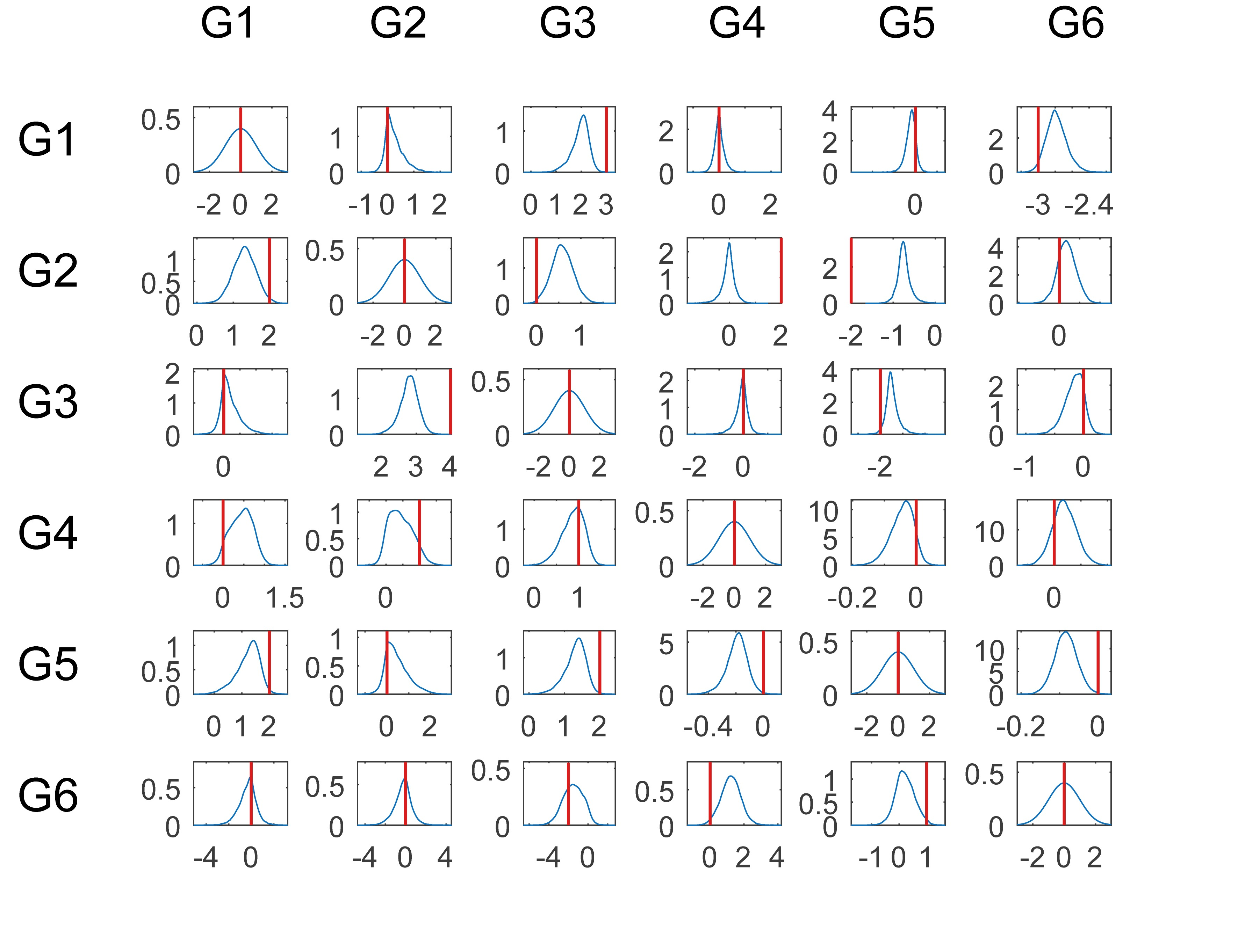

Supplement: S3 Fig — The sub-figure located in i-th row and j-th column represents the posterior distribution of the regulatory coefficient from gene j (Gj) to gene i (Gi). The red lines represent the parameter values of aij used for generating the ground-truth network as in Equation (S22). An interaction was viewed present if the k% credible interval for corresponding regulatory coefficient aij did not contain zero, otherwise absence. (TIF) [file pcbi.1008379.s003.tif]

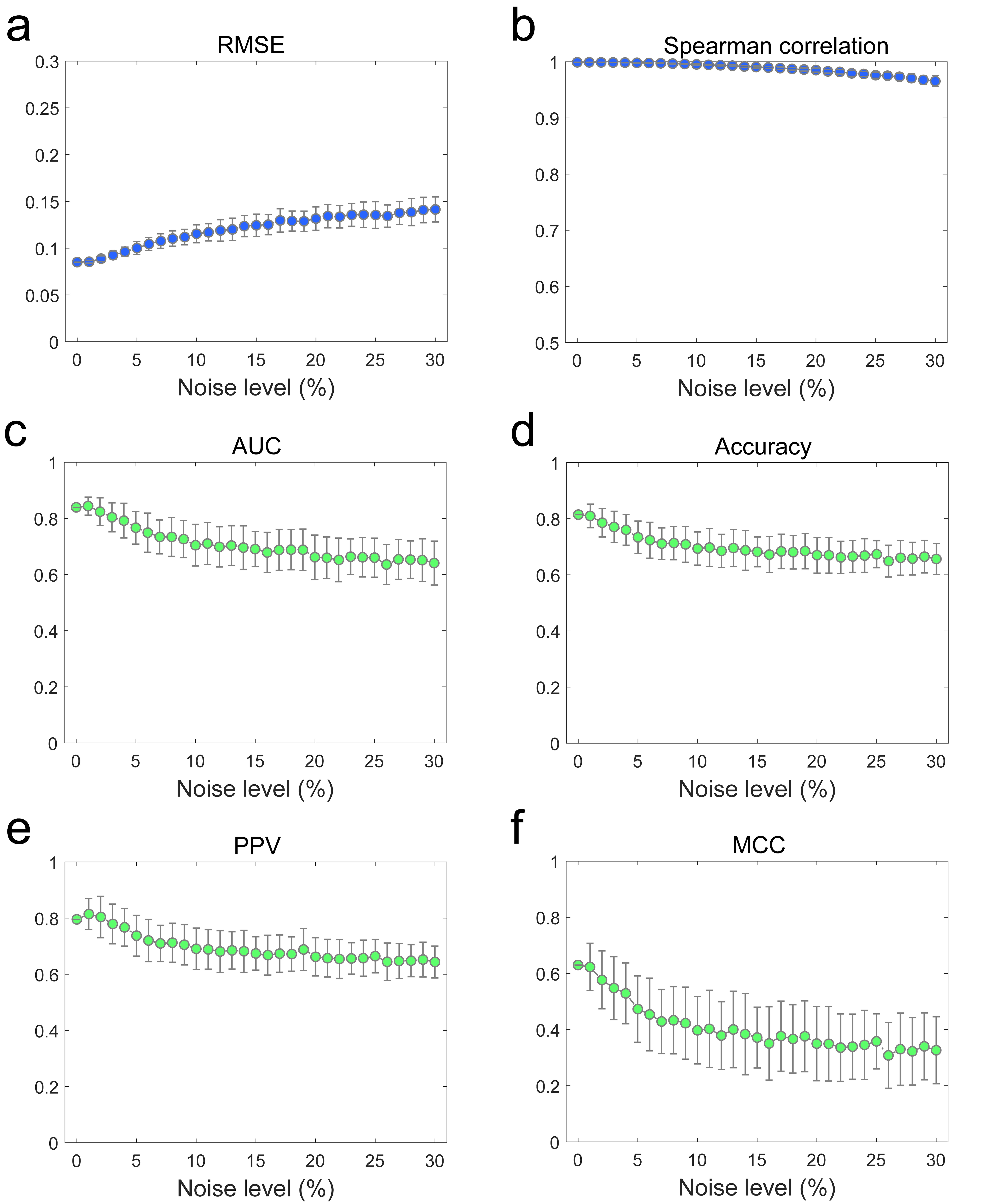

Supplement: S4 Fig — The levels of measurement variabilities in the synthetic data were quantified using the coefficient of variations (CVs) (from 0% to 30%). (a-b) Root of mean squared error (RMSE) and Spearman correlation used for evaluating the accuracy of the temporal progression inference. (c-f) AUC, accuracy rate, positive predictive rate (PPV) and Matthews correlation coefficient (MCC) used for evaluating the robustness of the GRN inference. (TIF) [file pcbi.1008379.s004.tif]

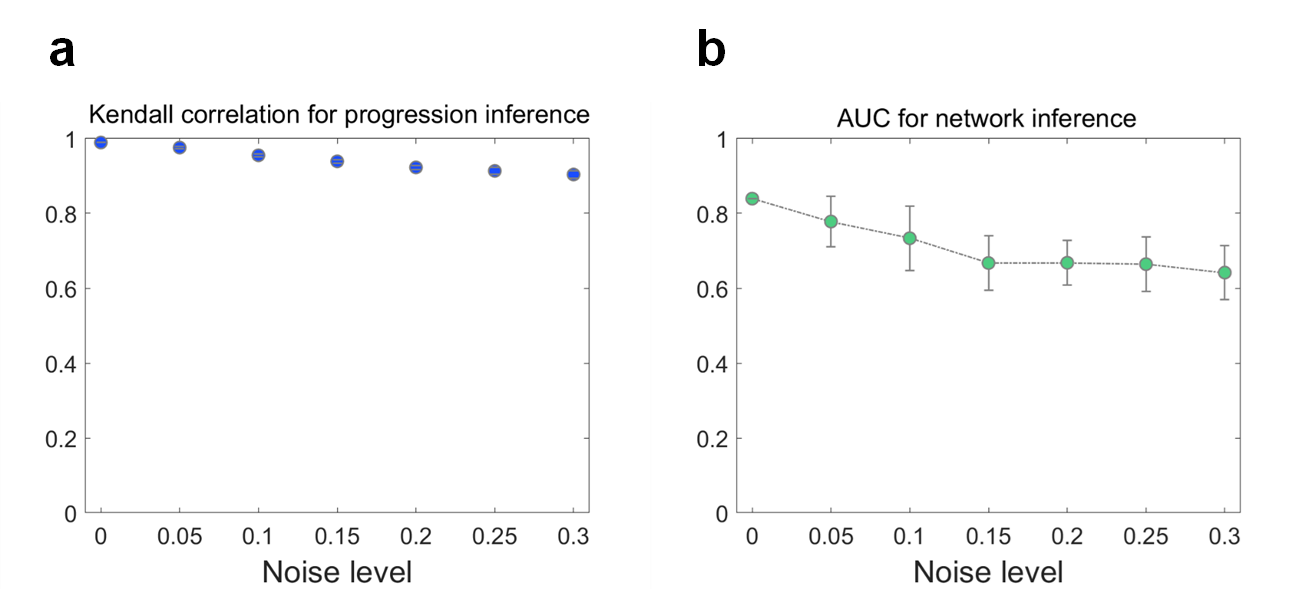

Supplement: S5 Fig — The noises were generated from the exponential distribution with mean ranging from 0 to 0.3. (a) Kendall correlation for evaluating the accuracy of the temporal progression inference. (b) AUC for evaluating the robustness of the GRN inference. (TIF) [file pcbi.1008379.s005.tif]

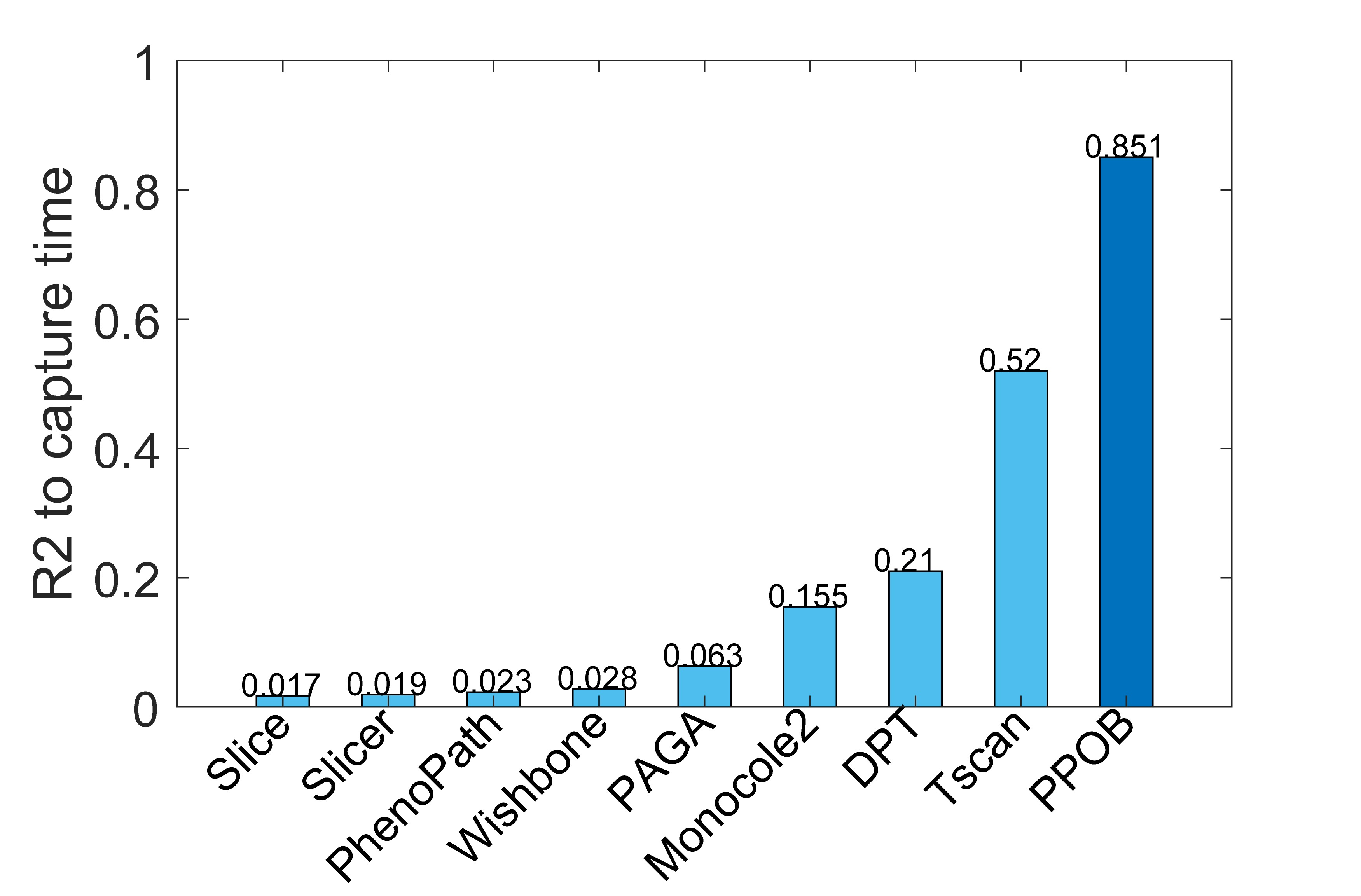

Supplement: S6 Fig — A set of scRNA-seq data of dendritic cells stimulated with LPS was used for benchmarking. The cells were sequenced at 1, 2, 4 and 6h after stimulation of LPS. We compared PROB with other pseudotime inference methods (Slice, Slicer, PhenoPath, Wishbone, PAGA, Monocole2, DPT, Tscan) in cell ordering. The coefficient of determination (i.e., R2) between the estimated pseudotime and the capture time of cells was used for evaluation. PROB outperformed the other existing methods. (TIF) [file pcbi.1008379.s006.tif]

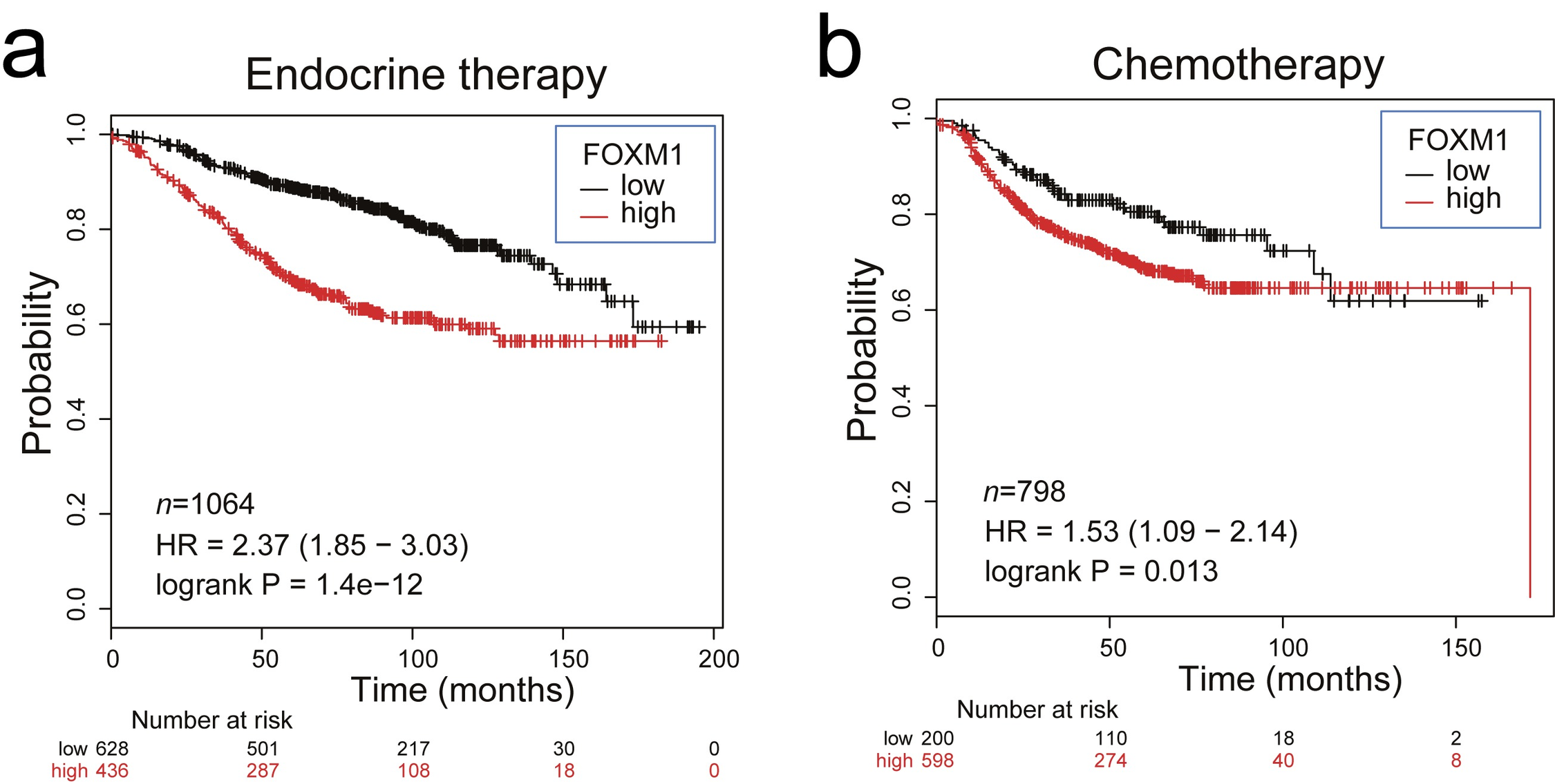

Supplement: S7 Fig — Breast cancer patients who received endocrine therapy (a) or chemotherapy (b) were included into the K-M survival analysis. Kaplan-Meier Plotter (http://kmplot.com) [67] was employed to perform analysis. Log-rank test p-value was used to assess the prognostic significance. (TIF) [file pcbi.1008379.s007.tif]

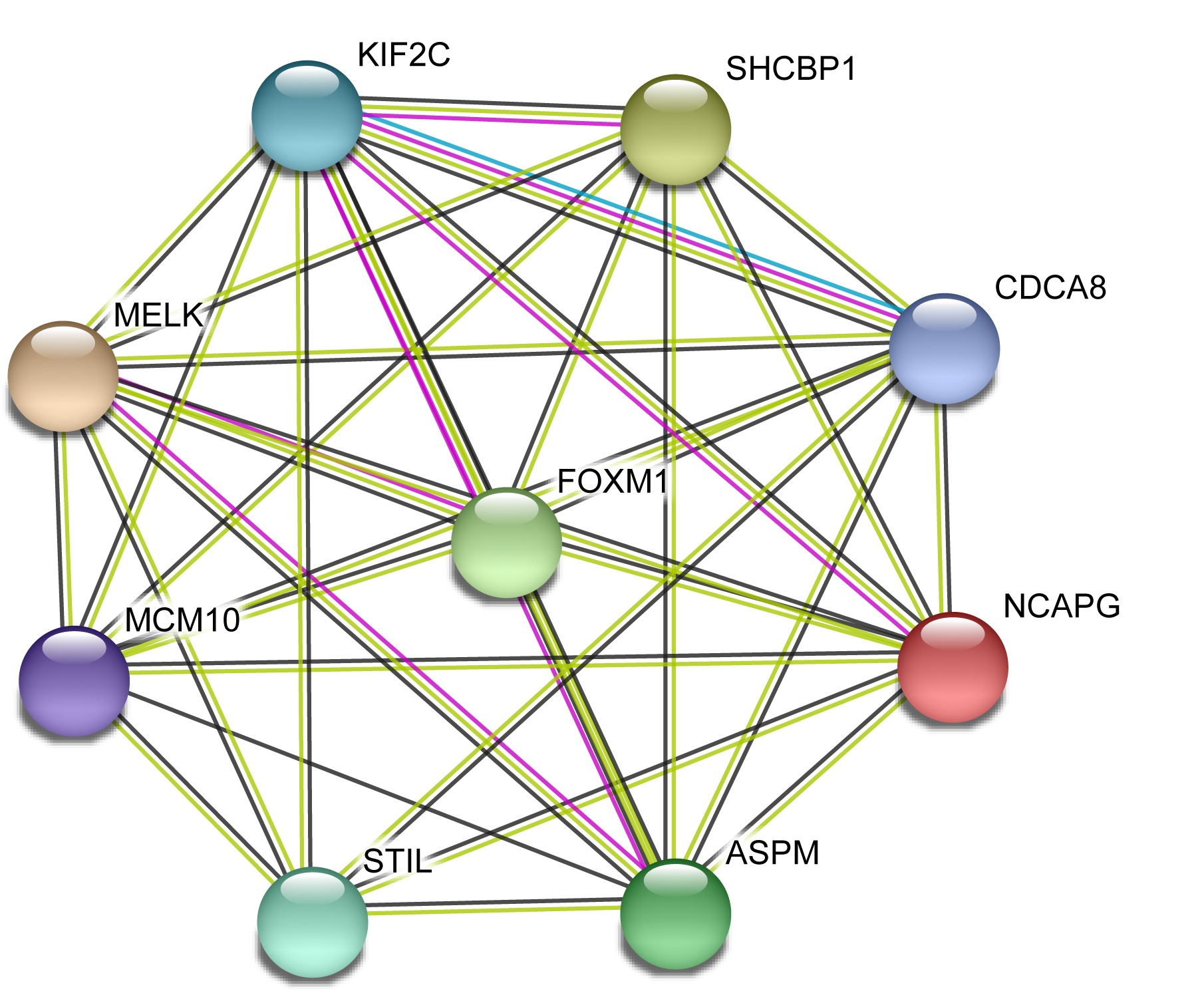

Supplement: S8 Fig — The network shows the co-expression or regulation between FOXM1 and the predicted targeted genes. Among 8 targeted genes of FOXM1 predicted from PROB, 7 genes (including KIF2C, SHCBP1, CDCA8, NCAPG, ASPM, MELK and MCM10) were supported by the database information. (TIF) [file pcbi.1008379.s008.tif]

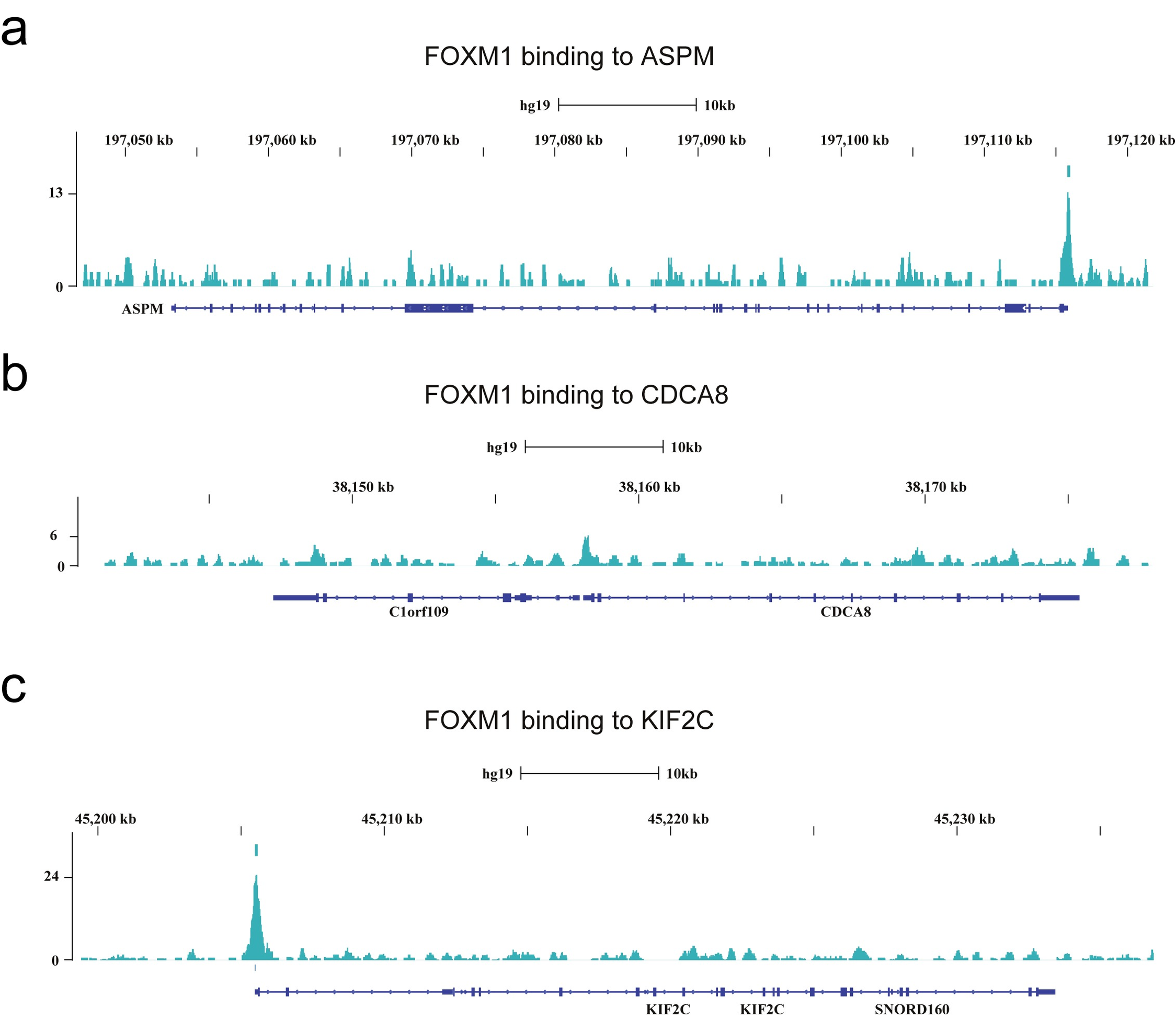

Supplement: S9 Fig — ChIP-seq data were downloaded from GEO database (GSE62425) [54]. The analysis results showed FOXM1 binds ASPM and KIF2C. (TIF) [file pcbi.1008379.s009.tif]

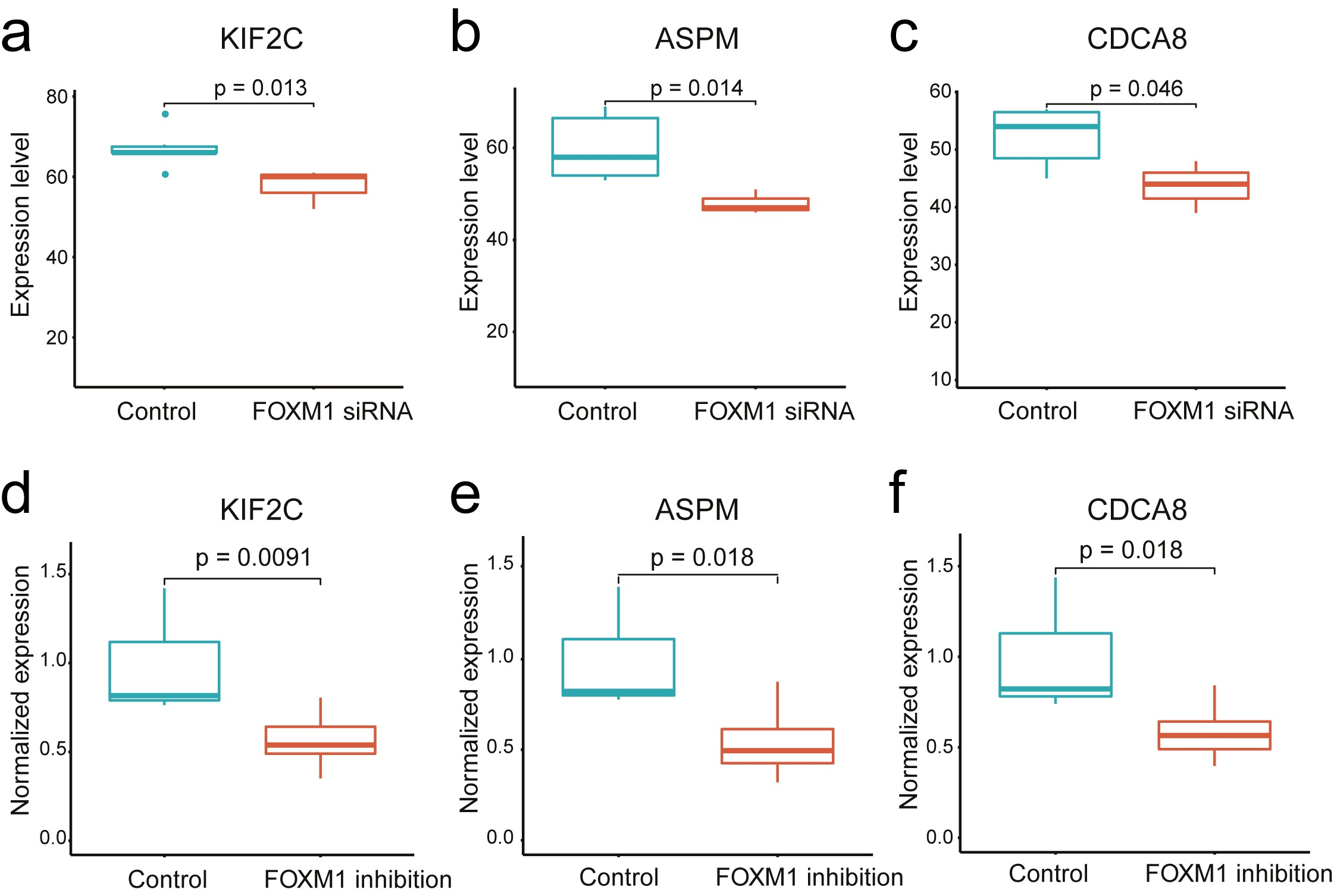

Supplement: S10 Fig — Microarray data and RNA-seq data on two breast cancer cell lines (BT-20 and MCF-7, respectively) were used for analyses. (a-c) The expression levels of the above three genes in BT-20 breast cancer cells under FOXM1 siRNA or control (mock transfection and GFP siRNA) conditions were analyzed using a set of microarray data (GSE2222) [55] (d-f) RNA-seq data (GSE58626) [56] of MCF-7 breast cancer cells was used to analyze the differential expressions of the above three genes after FOXM1 inhibition by using small molecule compound IB that specifically inhibits FOXM1 [56]. The knockdown or silence of FOXM1 significantly reduced the expressions of the above three genes. Wilcoxon rank sum test (one-tailed) p value was calculated to assess the statistical significance. (TIF) [file pcbi.1008379.s010.tif]

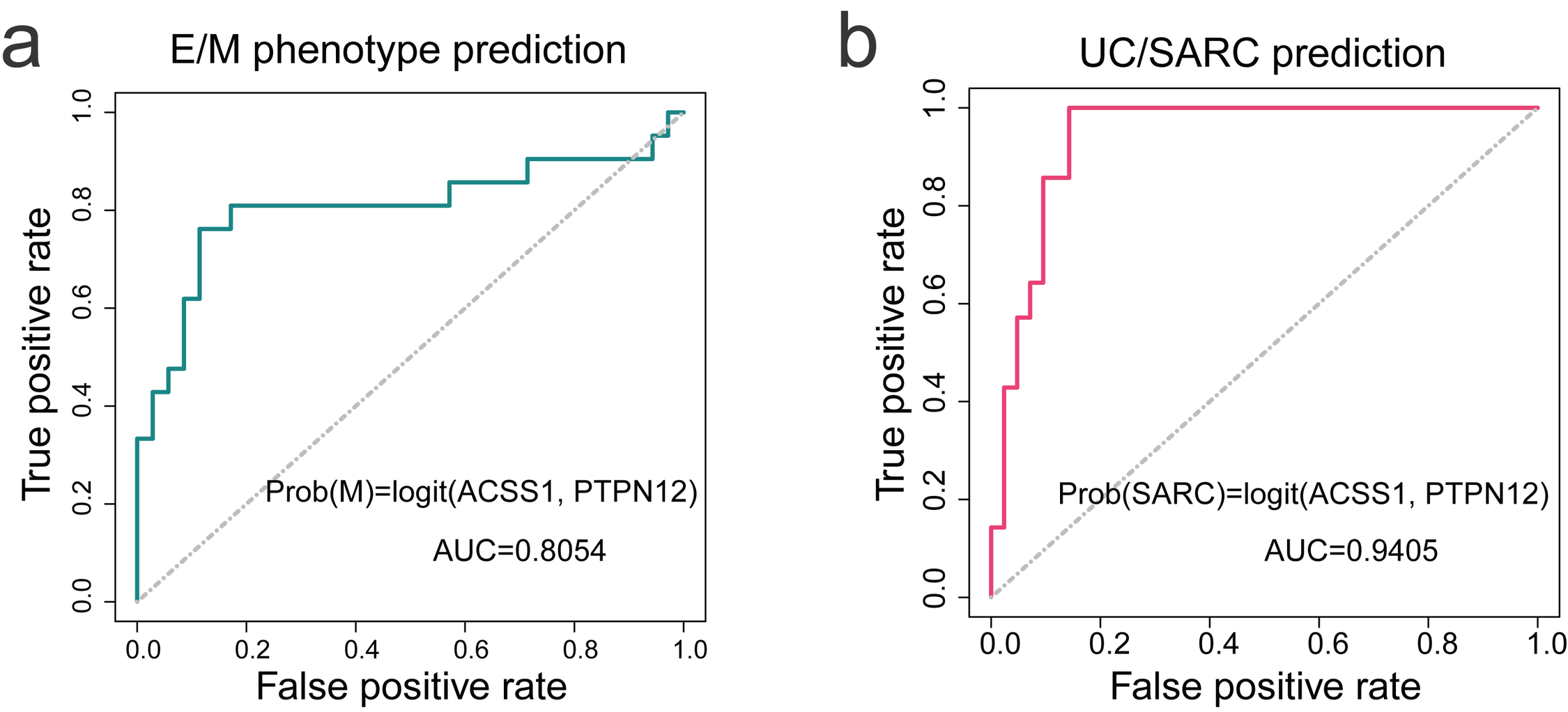

Supplement: S11 Fig — A logistic regression model was developed to predict (a) EMT states or (b) histological subtypes (UC vs. SARC) of bladder cancer based on the expression levels of ACSS1 and PTPN12. The samples were randomly divided into training set (n = 56) and test set (n = 56). The AUCs for the EMT phenotype prediction and subtype prediction are 0.8054 and 0.9405, respectively. (TIF) [file pcbi.1008379.s011.tif]

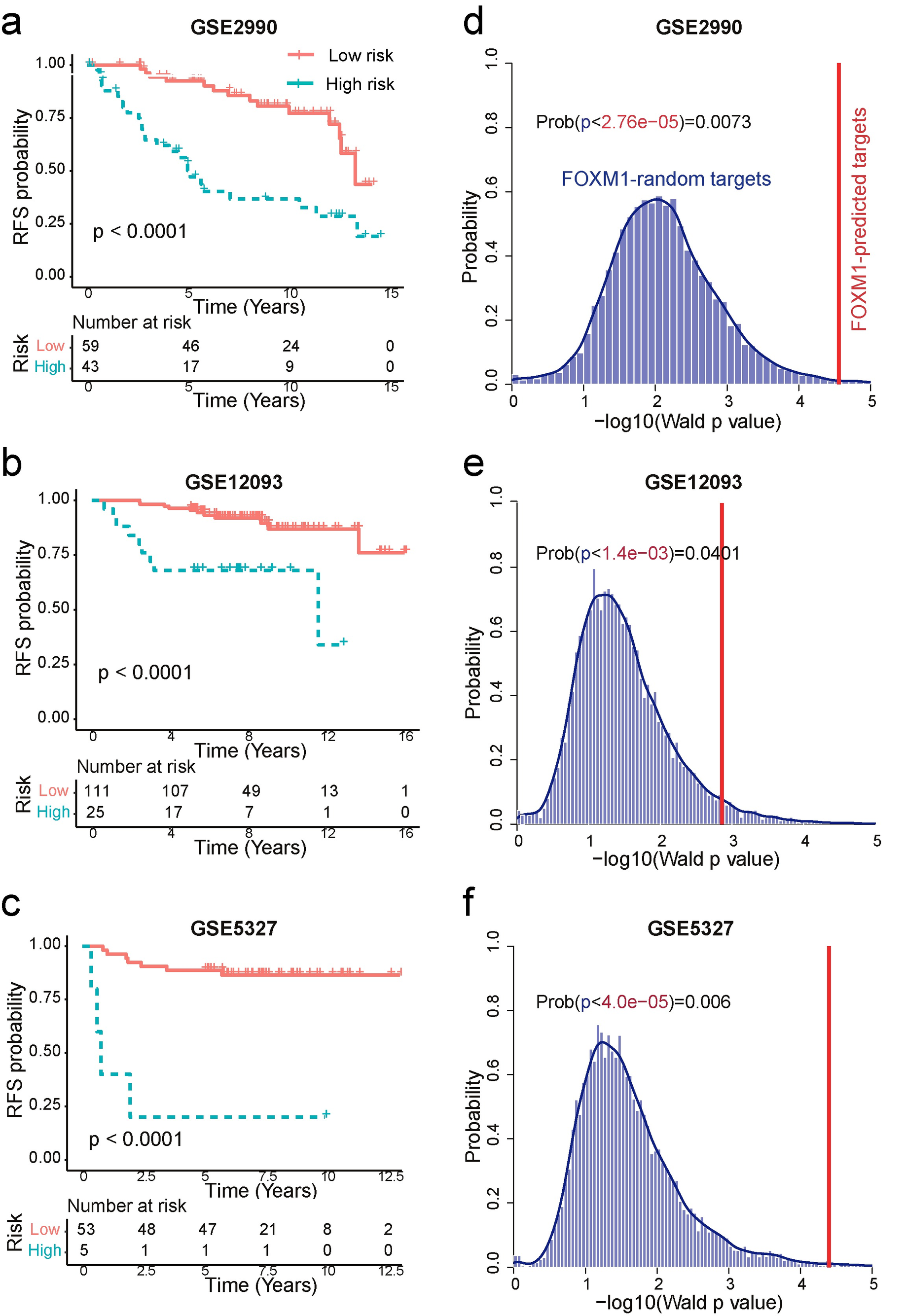

Supplement: S12 Fig — The kinetic features of the FOXM1-target interactions were formulated as a risk score to predict relapse for breast cancer patients in multiple independent cohorts. (a-c) Prognostic significance of the FOXM1-target interactions with respect to predicting relapse-free survival (RFS) in breast cancer evaluated on different datasets (GSE2990 [68], GSE12093 [69] and GSE5327 [70]). The log-rank test p value was used to assess the statistical significance of the difference between the Kaplan-Meier (K-M) survival curves of the high-risk group (green) and the low-risk group (red) of patients. (d-f) Nonrandomness test of the FOXM1-target interactions in predicting relapse in breast cancer using a bootstrapping approach (Text S6). The permutation test p values for all three datasets (0.0073, 0.0401 and 0.006, respectively) were less than 0.05, verifying the statistical significance of the prognostic power of the FOXM1-target interactions. (TIF) [file pcbi.1008379.s012.tif]
