# Supplementary material for: Inferring latent temporal progression and regulatory networks from cross-sectional transcriptomic data of cancer samples
Source: PLoS Comput Biol. 2021 Mar 5;17(3):e1008379. doi: 10.1371/journal.pcbi.1008379 (PMC7968745; doi:10.1371/journal.pcbi.1008379)
Supplement: S1 Table — (DOCX) [file pcbi.1008379.s013.docx]

**Table S1.** Out-degree values of the genes in the UC-specific and SARC-specific networks.

| **Gene name** | **UC-specific network** | **SARC-specific network** | **Pathways** |
| --- | --- | --- | --- |
| TGFB1 | 5 | 12 | TGFB1 pathway |
| PSMD1 | 0 | 2 |  |
| MSN | 0 | 2 |  |
| DNMT1 | 10 | 0 |  |
| **ACSS1** | 0 | **29** |  |
| CPT1B | 0 | 14 |  |
| WNT5B | 0 | 6 |  |
| RIN1 | 0 | 5 |  |
| GNRH1 | 23 | 0 |  |
| PMM1 | 5 | 21 |  |
| PI4KA | 0 | 1 | RhoA pathway |
| ARHGEF1 | 0 | 2 |  |
| PIP4K2B | 38 | 0 |  |
| PKN1 | 0 | 1 |  |
| GNA12 | 0 | 20 |  |
| MYL6B | 0 | 5 |  |
| CFL2 | 1 | 0 |  |
| PIKFYVE | 0 | 7 |  |
| Septin 2 | 0 | 5 |  |
| BTG2 | 0 | 3 |  |
| LGALS3 | 0 | 17 | p53 pathway |
| RFWD2 | 15 | 0 |  |
| TCF7L2 | 0 | 0 |  |
| FXYD3 | 3 | 1 |  |
| SH3BGRL2 | 0 | 5 |  |
| GNAI1 | 35 | 0 |  |
| PHLDA3 | 1 | 0 |  |
| F11R | 0 | 0 |  |
| SH3BP4 | 3 | 12 |  |
| TP63 | 0 | 3 | p63 pathway |
| CCNG1 | 0 | 3 |  |
| **PTPN12** | **40** | 0 |  |
| CSTA | 0 | 4 |  |
| PERP | 40 | 0 |  |
| PMAIP1 | 0 | 0 |  |
| MYC | 1 | 1 |  |
| RAC2 | 39 | 1 |  |
| KRT1 | 1 | 6 |  |
| SNAI2 | 0 | 13 | EMT transcriptional regulators/markers |
| SNAI3 | 0 | 3 |  |
| TWIST1 | 0 | 25 |  |
| CDH1 | 0 | 0 |  |
| TJP1 | 1 | 4 |  |
| CLDN1 | 0 | 3 |  |
