# Supplementary material for: Inferring latent temporal progression and regulatory networks from cross-sectional transcriptomic data of cancer samples
Source: PLoS Comput Biol. 2021 Mar 5;17(3):e1008379. doi: 10.1371/journal.pcbi.1008379 (PMC7968745; doi:10.1371/journal.pcbi.1008379)
Supplement: S2 Table — (DOCX) [file pcbi.1008379.s014.docx]

**Table S2.** The siRNA sequence used in this study.

| Si RNA Name | Sense (5’-3’) | Antisense (5’-3’) |
| --- | --- | --- |
| H-ACSS1-675 | GCAAGGUGGUUAUCACCUUTT | AAGGUGAUAACCACCUUGCTT |
| H-ACSS1-1040 | GCCGACAUCGGUUGGAUUATT | UAAUCCAACCGAUGUCGGCTT |
| H-ACSS1-1608 | CCUACCCAGGCUAUUACUUTT | AAGUAAUAGCCUGGGUAGGTT |
