# Supplementary material for: Inferring latent temporal progression and regulatory networks from cross-sectional transcriptomic data of cancer samples
Source: PLoS Comput Biol. 2021 Mar 5;17(3):e1008379. doi: 10.1371/journal.pcbi.1008379 (PMC7968745; doi:10.1371/journal.pcbi.1008379)
Supplement: S3 Table — (DOCX) [file pcbi.1008379.s015.docx]

**Table S3**. The specific primers used in this study.

| Primer Name | Sequence (5’-3’) |
| --- | --- |
| hGAPDH-qF | GGAGCGAGATCCCTCCAAAAT |
| hGAPDH-qR | GGCTGTTGTCATACTTCTCATGG |
| hACSS1-qF | TGGAGAGGCTATGCGACA |
| hACSS1-qR | TGGCTCAAGAGGGCAGA |
| hE-Cadherin-qF | CGAGAGCTACACGTTCACGG |
| hE-Cadherin-qR | GGGTGTCGAGGGAAAAATAGG |
