# Supplementary material for: Inferring latent temporal progression and regulatory networks from cross-sectional transcriptomic data of cancer samples
Source: PLoS Comput Biol. 2021 Mar 5;17(3):e1008379. doi: 10.1371/journal.pcbi.1008379 (PMC7968745; doi:10.1371/journal.pcbi.1008379)
Supplement: S4 Table — (DOCX) [file pcbi.1008379.s016.docx]

**Table S4.** Runtime of PROB on different datasets.

| **Datasets** | **Gene number** | **Sample number** | **Network size** | **Running time (**sec.**)** | | |
| --- | --- | --- | --- | --- | --- | --- |
|  |  |  |  | **Progression inference** | **Bayesian Lasso** | **Total** |
| DC-LPS scRNA-seq | 44847 | 479 | 23 | 21.18 | 19.37 | 40.55 |
| UC_SARC | 29377 | 112 | 44 | 1.13 | 56.88 | 58.01 |
| Breast cancer | 22282 | 196 | 100 | 1.95 | 57.47 | 59.42 |
