# Supplementary material for: Inferring latent temporal progression and regulatory networks from cross-sectional transcriptomic data of cancer samples
Source: PLoS Comput Biol. 2021 Mar 5;17(3):e1008379. doi: 10.1371/journal.pcbi.1008379 (PMC7968745; doi:10.1371/journal.pcbi.1008379)
Supplement: S1 Text — (DOCX) [file pcbi.1008379.s017.docx]

**Text S1. Method details of PROB**

**Progression-dependent dynamic modeling of the GRN**

Let $X_{i}(t,s)$ represent the expression level of gene *i* (*i* =1*,…,n*) at time *t* in cancer with progression status *s*. Assume cancer progression is an irreversible process over time so that is a strictly monotonic increasing function of *t*, i.e., . As such, has an inverse function , and .

The change rate of $X_{i}(t,s)$ after a small progression period $\Delta s$ can be modeled by the following difference equation, assuming mass action kinetics [1]:

| , | (S1) |
| --- | --- |

where $a_{ij}$ is the regulatory coefficient from gene *j* to gene *i* (*j*≠*i*), and $d_{i}$ is the self-degradation rate of gene *i*.

As $\Delta s\to0$, we obtain the following progression-structured model in the form of partial differential equations (PDEs),

| . | (S2) |
| --- | --- |

Since gene regulations or biochemical reactions are notably faster than cancer progression, we could assume that, in the above equations, $X_{i}(t,s)$ quickly approaches its steady-state $\bar{X}_{i}(s)$ as *s* changes, that is, $\frac{\partial\bar{X}_{i}(s)}{\partial t}=0$. Therefore, we have the following ordinary differential equations (ODEs) for the GRN:

| $\frac{d\bar{X}_{i}(s)}{ds}=\frac{\partial\bar{X}_{i}(s)}{\partial s}=\sum_{j=1}^{n} a_{ij}\bar{X}_{i}(s)\cdot\bar{X}_{j}(s)-d_{i}\bar{X}_{i}(s)$. | (S3) |
| --- | --- |

For simplicity, in the following sections, we write $\bar{X}_{i}(s)$ as $X_{i}(s)$ when there is no ambiguity. Equation (S3) can therefore also read

| $\frac{dX_{i}(s)}{ds}=\sum_{j=1}^{n} a_{ij}X_{i}(s)\cdot X_{j}(s)-d_{i}X_{i}(s)$. | (S4) |
| --- | --- |

**Supplementary references**

1. Chan TE, Stumpf MPH, Babtie AC. Gene Regulatory Network Inference from Single-Cell Data Using Multivariate Information Measures. Cell systems. 2017;5(3):251-67.e3. Epub 2017/09/29. doi: 10.1016/j.cels.2017.08.014. PubMed PMID: 28957658; PubMed Central PMCID: PMCPMC5624513.
