# Supplementary material for: Inferring latent temporal progression and regulatory networks from cross-sectional transcriptomic data of cancer samples
Source: PLoS Comput Biol. 2021 Mar 5;17(3):e1008379. doi: 10.1371/journal.pcbi.1008379 (PMC7968745; doi:10.1371/journal.pcbi.1008379)
Supplement: S2 Text — (DOCX) [file pcbi.1008379.s018.docx]

**Text S2. Proof of the Theorem 1**

***Theorem 1*** Assume there are two trajectories of latent-temporal progression, $s(r)$ and $\tilde{s}(r)$ with the same roots, $r\in I=[0,1]$. Define $\left\|\tilde{s}-s\right\|_{L^{2}}=\left(\int_{I} \left|\tilde{s}-s\right|^{2}dr\right)^{1/2}$. If $\left(X_{i}(s),a_{ij}\right)$ and $\left(X_{i}(\tilde{s}),\tilde{a}_{ij}\right)$ both satisfy the equations of progression-dependent dynamic model, i.e.,

| $\frac{dX_{i}(s)}{ds}=\sum_{j\neq i} a_{ij}X_{i}(s)\cdot X_{j}(s)-d_{i}X_{i}(s)$, $i=1,\cdots,n$, | (S5) |
| --- | --- |
| $\frac{dX_{i}(\tilde{s})}{d\tilde{s}}=\sum_{j\neq i} \tilde{a}_{ij}X_{i}(\tilde{s})\cdot X_{j}(\tilde{s})-\tilde{d}_{i}X_{i}(\tilde{s})$, $i=1,\cdots,n$, | (S6) |

then we have

| $\lim_{\left\Vert\tilde{s}-s\right\Vert\to0} \sum_{i,j=1}^{n} \left\vert\tilde{a}_{ij}-a_{ij}\right\vert^{2}=0$. | (S7) |
| --- | --- |

**Proof**: (*Step 1*). We first transform the Equation (S5) into the following form:

| $\frac{d\left(\log X_{i}(s)\right)}{ds}=\sum_{j\neq i} a_{ij}X_{j}(s)-d_{i}$, | (S8) |
| --- | --- |

Denoting $a_{ii}=0$ and integrating the above equation leads to

| $\log X_{i}(s)=\sum_{j=1}^{n} a_{ij}\int_{0}^{s} X_{j}(\tau)d\tau-d_{i}\cdot s+\log X_{i}(0)$, | (S9) |
| --- | --- |

Denote $Y_{i}\left(s\right)=\log X_{i}(s)$, $Z_{j}(s)=\int_{0}^{s} X_{j}(\tau)d\tau$ $\left(j=1,2,\cdots,n;\text{ }j\neq i\right)$ . The above equation can be written as

| $\begin{matrix} Y_{i}(s)=\sum_{j=1}^{n} a_{ij}Z_{j}(s)-d_{i}s+Y_{i}(0) \\ \text{ }=\sum_{j=1}^{n} \left(a_{ij}-\tilde{a}_{ij}\right)Z_{j}(s)+\sum_{j=1}^{n} \tilde{a}_{ij}Z_{j}(s)-d_{i}s+Y_{i}(0) \\ \text{ }=\sum_{j=1}^{n} \left(a_{ij}-\tilde{a}_{ij}\right)Z_{j}(s)+\left[\sum_{j=1}^{n} \tilde{a}_{ij}Z_{j}(\tilde{s})-\tilde{d}_{i}\tilde{s}+Y_{i}(0)\right]+\sum_{j=1}^{n} \tilde{a}_{ij}\left(Z_{j}(s)-Z_{j}(\tilde{s})\right)+\tilde{d}_{i}\tilde{s}-d_{i}s \\ \text{ }=\sum_{j=1}^{n} \left(a_{ij}-\tilde{a}_{ij}\right)Z_{j}(s)+Y_{i}(\tilde{s})+\sum_{j=1}^{n} \tilde{a}_{ij}\left(Z_{j}(s)-Z_{j}(\tilde{s})\right)-\tilde{d}_{i}\cdot\left(s-\tilde{s}\right)-\left(d_{i}-\tilde{d}_{i}\right)\cdot s \end{matrix}$. | (S10) |
| --- | --- |

Therefore,

| $\sum_{j=1}^{n} \left(a_{ij}-\tilde{a}_{ij}\right)Z_{j}(s)\text{+}\left(d_{i}-\tilde{d}_{i}\right)\cdot s=Y_{i}(s)-Y_{i}(\tilde{s})-\sum_{j=1}^{n} \tilde{a}_{ij}\left(Z_{j}(s)-Z_{j}(\tilde{s})\right)-\tilde{d}_{i}\cdot(s-\tilde{s})$. | (S11) |
| --- | --- |

For $i,j=1,2,\cdots,n$, define $w_{ij}=\left\{\begin{matrix} a_{ij}-\tilde{a}_{ij},\text{ }i\neq j \\ d_{i}-\tilde{d}_{i}\text{ },\text{ }i=j \end{matrix}\right.$,

and redefine $Z_{j}(s)=\left\{\begin{matrix} \int_{0}^{s} X_{j}(\tau)d\tau,\text{ }i\neq j \\ s,\text{ }i\text{=}j \end{matrix}\right.$.

Denote $W=\left(w_{ij}\right)_{n\times n}$, and $W_{i}=\left(w_{i1},w_{i2},\cdots,w_{in}\right)_{1\times n}$ is the *i*-th row of *W.* Also denote $Z=\left(Z_{1}(s),Z_{2}(s),\ldots,Z_{n}(s)\right)^{T}$, and $V_{i}=Y_{i}(s)-Y_{i}(\tilde{s})-\sum_{j=1}^{n} \tilde{a}_{ij}\left(Z_{j}(s)-Z_{j}(\tilde{s})\right)-\tilde{d}_{i}\cdot(s-\tilde{s})$. The Equation (S11) can be written as

| $W_{i}Z=V_{i}$. | (S12) |
| --- | --- |

(*Step 2*). Take *m* points on $s\left(r\right)$ and $\tilde{s}\left(r\right)$, respectively: $S_{i}=s\left(r_{i}\right)$, $\tilde{S}_{i}=\tilde{s}\left(r_{i}\right)$, where $r_{i}=i/m$,$i=0,1,\cdots,m$.

Let $T=\left(t_{ij}\right)_{n\times m}=\left(Z_{i}(S_{j})\right)_{n\times m}$, $U_{i}=\left(V_{i}(r_{1}),\cdots,V_{i}(r_{m})\right)^{T}_{1\times m}$. Then we have

| $TW_{i}=U_{i}$. | (S13) |
| --- | --- |

Therefore,

| $\left(a_{ij}-\tilde{a}_{ij}\right)_{\cdot,j}=\hat{W}_{i}=\mathop{\mathrm{argmin}}\limits_{W_{i}} \left\Vert U_{i}-TW_{i}\right\Vert^{2}=\mathop{\mathrm{argmin}}\limits_{W_{i}} \left(U_{i}-TW_{i}\right)^{T}\cdot\left(U_{i}-TW_{i}\right)$. | (S14) |
| --- | --- |

Via matrix derivation, we get:

(a) when *T* is n-full rank,

| $\hat{W}_{i}=\left(T^{T}T\right)^{-1}T^{T}U_{i}$. | (S15) |
| --- | --- |

(b) when *T* is not n-full rank, there exists $\lambda>0$ such that

| $\hat{W}_{i}=\left(T^{T}T+\lambda_{i}I\right)^{-1}T^{T}U_{i}$. | (S16) |
| --- | --- |

(*Step 3*). From the definition of $W_{i}$ and Equations (S15-S16), we know that

| $\sum_{j=1}^{n} \left(a_{ij}-\tilde{a}_{ij}\right)^{2}+\left(d_{i}-\tilde{d}_{i}\right)^{2}\leq C\cdot\left\Vert U_{i}\right\Vert_{L^{2}}^{2}$, | (S17) |
| --- | --- |

where *C* is a positive constant. According to the definition of *U_i_*, we have

| $\left\Vert U_{i}\right\Vert_{L^{2}}\leq C\cdot\left\Vert V_{i}\right\Vert_{L^{2}}\leq\left\Vert Y_{i}(s)-Y_{i}(\tilde{s})\right\Vert_{L^{2}}+\left\Vert\sum_{j=1}^{n} \tilde{a}_{ij}\left(Z_{j}(s)-Z_{j}(\tilde{s})\right)\right\Vert_{L^{2}}+\tilde{d}_{i}\cdot\left\Vert s-\tilde{s}\right\Vert_{L^{2}}$, | (S18) |
| --- | --- |

We only need to prove that the first two terms in the right hand of the above inequality tends to 0 as $\left\|\tilde{s}-s\right\|\to0$.

(i) We now prove$\lim_{\left\|\tilde{s}-s\right\|\to0} \left\|Y_{i}(s)-Y_{i}(\tilde{s})\right\|_{L^{2}}=0$. Since $Y_{i}\left(s\right)=\log X_{i}(s)$, we only need to prove $\lim_{\left\|\tilde{s}-s\right\|\to0} \left\|X_{i}(s)-X_{i}(\tilde{s})\right\|_{L^{2}}=0$, which is valid according to the continuity of $X_{i}(s)$ with respect to *s*.

(ii) We next prove $\lim_{\left\|\tilde{s}-s\right\|\to0} \left\|\sum_{j=1}^{n} \tilde{a}_{ij}\left(Z_{j}(s)-Z_{j}(\tilde{s})\right)\right\|_{L^{2}}=0$.

| $\begin{matrix} \text{ }\sum_{j=1}^{n} \tilde{a}_{ij}\left(Z_{j}(s)-Z_{j}(\tilde{s})\right) \\ =-\sum_{j=1}^{n} \tilde{a}_{ij}\left(\int_{0}^{\tilde{s}} \tilde{X}_{j}(\tau)d\tau-\int_{0}^{s} X_{j}(\tau)d\tau\right) \\ =-\sum_{j=1}^{n} \tilde{a}_{ij}\left[\lim_{N\to\infty} \sum_{l=0}^{N} \left(\int_{\tilde{S}_{l}}^{\tilde{S}_{l+1}} \tilde{X}_{j}(\tau)d\tau-\int_{S_{l}}^{S_{l+1}} X_{j}(\tau)d\tau\right)\right]\text{, (}N=m-1\text{)} \end{matrix}$ | (S19) |
| --- | --- |

For any *N*, since $\left\|\tilde{s}-s\right\|\to0$, so $\tilde{S}_{l}\to S_{l}$ ($l=0,1,\cdots,N+1$), and thus

| $\begin{matrix} \text{ }\lim_{\left\Vert\tilde{s}-s\right\Vert\to0} \sum_{l=0}^{N} \left(\int_{\tilde{S}_{l}}^{\tilde{S}_{l+1}} \tilde{X}_{j}(\tau)d\tau-\int_{S_{l}}^{S_{l+1}} X_{j}(\tau)d\tau\right) \\ =\sum_{l=0}^{N} \lim_{\left\Vert\tilde{s}-s\right\Vert\to0} \left(\int_{\tilde{S}_{l}}^{\tilde{S}_{l+1}} \tilde{X}_{j}(\tau)d\tau-\int_{S_{l}}^{S_{l+1}} X_{j}(\tau)d\tau\right)=0. \end{matrix}$ | (S20) |
| --- | --- |

That is, the above limit is uniformly convergent with respect to N. Therefore,

| $\begin{matrix} \text{ }\lim_{\left\Vert\tilde{s}-s\right\Vert\to0} \left(\int_{0}^{\tilde{s}} \tilde{X}_{j}(\tau)d\tau-\int_{0}^{s} X_{j}(\tau)d\tau\right) \\ =\lim_{\left\Vert\tilde{s}-s\right\Vert\to0} \lim_{N\to\infty} \sum_{l=0}^{N} \left(\int_{\tilde{S}_{l}}^{\tilde{S}_{l+1}} \tilde{X}_{j}(\tau)d\tau-\int_{S_{l}}^{S_{l+1}} X_{j}(\tau)d\tau\right) \\ =\lim_{N\to\infty} \sum_{l=0}^{N} \lim_{\left\Vert\tilde{s}-s\right\Vert\to0} \left(\int_{\tilde{S}_{l}}^{\tilde{S}_{l+1}} \tilde{X}_{j}(\tau)d\tau-\int_{S_{l}}^{S_{l+1}} X_{j}(\tau)d\tau\right) \\ =0 \end{matrix}$ | (S21) |
| --- | --- |

From the above Equations (S19-S21), we obtain that $\lim_{\left\|\tilde{s}-s\right\|\to0} \left\|\sum_{j=1}^{n} \tilde{a}_{ij}\left(Z_{j}(s)-Z_{j}(\tilde{s})\right)\right\|_{L^{2}}=0$.

Therefore, take the above steps (i) and (ii) and Equations (S17-S18) together, we have proved that the Equation (S7) holds.

Proof done.

□
