# Supplementary material for: Inferring latent temporal progression and regulatory networks from cross-sectional transcriptomic data of cancer samples
Source: PLoS Comput Biol. 2021 Mar 5;17(3):e1008379. doi: 10.1371/journal.pcbi.1008379 (PMC7968745; doi:10.1371/journal.pcbi.1008379)
Supplement: S3 Text — (DOCX) [file pcbi.1008379.s019.docx]

**Text S3. Implementation of PROB**

PROB was implemented in Matlab. The script of PROB contains two main executable functions, ‘Progression_Inferrence’ and ‘ODE_BayesianLasso’, for inferring latent-temporal progression and inferring ODE network using Bayessian Lasso, respectively. The codes are available at https://github.com/dongbusun/PROB.

**1. Latent-temporal progression inference**

[Data_ordered,TPD, TimeSampled]=Progression_Inferrence(Data)

This function (.m) was designed to infer latent-temporal progression from the cross-sectional gene expression data.

*Input*:

Data: a *n*×*m* matrix containing gene expression profiles (the first *n*-1 rows) and stage information of patients (the last row). *m* is sample size, i.e., the number of patients.

*Output*:

Data_ordered: a matrix containing the ordered (and smoothed) gene expression data;

TPD: a vector containing temporal progression distance for each patient;

TimeSampled: a vector containing standardized time-points sampled for Data_ordered.

**2. Bayesian Lasso for GRN parameter estimation**

[Para_Post_pdf,S,AM]=ODE_BayesianLasso(Data_ordered, TimeSampled)

This function (.m) was designed to reconstruct causal GRN based on the results of the above latent-temporal progression inference.

*Input*:

Data_ordered: matrix for the ordered (and smoothed) gene expression data (i.e., a subset from the output of the first function);

TimeSampled: vector for the standardized time-points associated with Data_ordered (i.e., the output of the first function).

*Output*:

Para_Post_pdf: cell format saving the posterior distribution over the regulatory coefficients of each gene in the GRN model.

S: a matrix saving the presence probability.

AM: Adjacent matrix of the inferred GRN. (*a_ij_*) for the regulatory strength from gene *j* to gene *i*.

In this function, the Matlab function ‘*bayeslm*’ and ‘*estimate*’ were, respectively, used to build the prior model and to estimate the posterior distributions of $A_{i}$ and $\sigma_{i}^{2}$. A custom implementation was performed to impose prior constrain on *diag*(*A*) by setting each *a_ii_* $(i=1,2,\cdots,n)$ to 0, or equivalently, by removing the *i*-th row of $X^{(i)}$ when passing it to the algorithm. In addition, an initial estimation of $A_{i}$ using LASSO regression via 10-fold cross validation was adopted as the value of parameter '*BetaStart*' in the function ‘*estimate*’.

**3. Visualization**

1) Progression_Plot(Data)

This function (.m) is designed to plot latent-temporal progression trajectory.

*Input*:

Data: a *n*×*m* matrix containing gene expression profiles (the first *n*-1 rows) and stage information of patients (the last row). *m* is sample size, i.e., the number of patients.

*Output*:

Two figures of latent-temporal progression trajectory and latent-temporal progression score along the ordering of patients.

2) TemporalGene_Plot(geneID,TimeSampled,Data_ordered)

This function (.m) is designed to plot latent-temporal expression of the selected genes.

*Input*:

geneID: the ID of genes selected for visualization.

Data_ordered: matrix for the ordered and smoothed gene expression data (i.e., the output of the first function);

TimeSampled: vector for the standardized time-points associated with Data_ordered (i.e., the output of the first function).

*Output*:

A figure of the expression dynamics of the selected genes over the inferred latent-temporal progression.

3) Cytoscape_Reformat(AM,NodeID)

This function (.R) was designed to reformat the AM to a suitable format for input as cytoscape software.

*Input*:

AM: The adjacent matrix of the inferred GRN resulted from the above function ‘ODE_BayesianLasso’.

NodeID: The ID or the symbol of the genes in the AM.

*Output*:

A matrix containing 3 columns: source nodes, interaction coefficients and target nodes.

**4. Incorporating prior network information**

[Para_Post_pdf,S,AM]=ODE_BayesianLasso_PriorNet(Data_ordered,TimeSampled,PriorNet)

This function (.m) was designed to incorporate prior network information based on mutual information into function ‘ODE_BayesianLasso’, when PROB is applied to large scale network reconstruction.

*Input*:

Data_ordered: matrix for the ordered and ordereded gene expression data (i.e., the output of the first function);

TimeSampled: vector for the standardized time-points associated with Data_ordered (i.e., the output of the first function).

PriorNet: a matrix for the prior network information. (*P_ij_*) represents the regulatory strength from gene *j* to gene *i*.

*Output*:

Para_Post_pdf: cell format saving the posterior distribution over the regulatory coefficients of each gene in the GRN model.

S: a matrix saving the presence probability.

AM: Adjacent matrix of the inferred GRN. (*a_ij_*) for the regulatory strength from gene *j* to gene *i*.
