# Supplementary material for: Inferring latent temporal progression and regulatory networks from cross-sectional transcriptomic data of cancer samples
Source: PLoS Comput Biol. 2021 Mar 5;17(3):e1008379. doi: 10.1371/journal.pcbi.1008379 (PMC7968745; doi:10.1371/journal.pcbi.1008379)
Supplement: S4 Text — (DOCX) [file pcbi.1008379.s020.docx]

**Text S4. Simulation study**

To assess the effectiveness and accuracy of PROB, we synthesized a GRN that was consisted of *n*=6 nodes and structured with typical motifs, such as positive and negative feedback loops and crosstalks (as shown in the following figure).

Under the same assumption of Equation (10) in the main text, the following ODEs were built to generate the gene expression changes along with the cancer progression,

| $\frac{dx_{i}}{ds}=\sum_{j=1}^{n} a_{ij}x_{i}x_{j}-d_{i}x_{i}$, $(i=1,2,\cdots,6)$. | (S22) |
| --- | --- |

The interaction confidents (*a_ij_*) were given by:

| $\left(a_{ij}\right)_{6\times6}=\left[\begin{matrix} \text{0 0 3 0 0 -3} \\ \text{2 0 0 2 -2 0} \\ \text{0 4 0 0 -2 0} \\ \text{0 1 1 0 0 0} \\ \text{2 0 2 0 0 0} \\ \text{0 0 -2 0 1 0} \end{matrix}\right]$. | (S23) |
| --- | --- |

The degradation rates (*d_i_*) were set as follows: (-1, -0.5, -1, -0.1, -1, -1). The initial value of each gene expression level in the above ODEs was set as standardized 1. By numerically solve the above ODEs we obtain a set of time series data. We uniformly sampled data to mimic gene expression profile of 100 patients ordered along with progression status (**Fig S1a**). The IDs of samples were then randomized to represent a set of transcriptomic data of cancer patients, assuming that their progression statuses were unknown (**Fig S1b**).

Based on the sample-randomized data, we evaluated whether the proposed method could recover the progression ordering and gene expression dynamics, and whether it could reconstruct the gene regulatory network (GRN).

We first used the developed graph-based random walk method to quantify the temporal progression distance of patients to the inferred ‘root’ in a simulated cohort. The inferred progression score of each patient was compared to the true progression. Along with the inferred progression trajectory, the course of gene expression was constructed. Subsequently, the GRN was reconstructed using Bayesian Lasso method as describe above.

An interaction was viewed present if the *k*% credible interval (CI) for corresponding interaction coefficient *a_ij_* did not contain zero, otherwise absence. We defined the following score to quantify the presence probability of each predicted interaction from gene *j* to gene *i*,

| $S_{ij}=1-\inf\left(k\left\vert0\notin CI_{k}\left(a_{ij}\right)\right.\right)$, | (S24) |
| --- | --- |

where $CI_{k}\left(a_{ij}\right)$ is the *k*% CI of posterior distribution of *a_ij_*.

The area under the receiver operator curve (AUC ROC) for presence/absence of interactions was used as a metric to evaluate the effectiveness of our method for recovering the network structure. True positive rate (TPR) and false positive rate (FPR) for the inferred network compared to the ground-truth network are defined by the following equations, respectively:

| $TPR=\frac{TP}{TP+FN}$, | (S25) |
| --- | --- |
| $FPR=\frac{FP}{FP+TN}$, | (S26) |

where TP, FP, TN and FN are the numbers of true positives, false positives, true negatives and false negatives, respectively. TPR and FPR were used to plot the receiver operating characteristic (ROC) curves. We used the trapezoidal method for calculating the area under curve (AUC) of ROC.

We also used additional evaluation metrics to evaluate the accuracy of the progression inference and GRN inference against a series of variability levels (with coefficient of variations (CVs) from 0% to 30%). Root of mean squared error (RMSE) and Spearman correlation were employed to evaluate the accuracy of the temporal progression inference; AUC, accuracy rate, positive predictive rate (PPV) and Matthews correlation coefficient (MCC) were employed to evaluate the accuracy of the GRN inference.
