# Supplementary material for: Inferring latent temporal progression and regulatory networks from cross-sectional transcriptomic data of cancer samples
Source: PLoS Comput Biol. 2021 Mar 5;17(3):e1008379. doi: 10.1371/journal.pcbi.1008379 (PMC7968745; doi:10.1371/journal.pcbi.1008379)
Supplement: S5 Text — (DOCX) [file pcbi.1008379.s021.docx]

**Text S5. PROB applied to real datasets**

***Gene selection for network inference***

In practice, one may encounter two cases of GRN inference: inferring GRN for a set of functionally related genes; or inferring GRN for a set of newly selected genes from the data. So, the genes for network construction could be selected according to prior biological knowledge or selected from the data for the specific purpose.

In the first example, we applied PROB to an expression dataset of bladder cancer (GSE128192) to reconstruct the regulatory relationships of the 44 EMT-regulatory genes [1] and investigate the network rewiring during EMT.

In the second example, PROB was applied to a microarray dataset of breast cancer (GSE7390) [2] to identify prognostic biomarker genes. Owing to the unique function of PROB in temporal progression inference and ordering of patients, the gene that most likely exhibits sustained increasing or decreasing patterns is competent as potential prognostic biomarker. So, we employed a trend analysis technique to select such genes based on smoothed expression data of patients ordered by progression scores, denoted as *X_i_*(*s_k_*), where *i* for gene index and *s_k_* for the ordered progression score. More specifically, the algorithm can be described as follows:

(i) We fitted each *X_i_* to a linear function. The estimated coefficient was denoted as *L_i_* and the constant was denoted as *C_i_*.

(ii) We then calculated the standard deviation of the detrended expression data of each gene, denoted as *V_i_*. The detrended data *H_i_*(*s_k_*) was obtained as *H_i_*(*s_k_*) = *X_i_*(*s_k_*)$- ($*L_i_*×*s_k_* + *C_i_*). Therefore, *V_i_*=*std*(*H_i_*(*s_k_*)).

(iii) A score was defined for each gene, *R_i_*= *|L_i_* /*V _i_*|.

(iv) The 100 top-ranked genes according to *R_i_* were selected.

The above selected genes are referred to as temporally changing genes (TCGs) in this study. Note that the TCGs selected by PROB are not equivalent to common highly-variable genes (HVGs). In fact, in the case study of breast cancer, the 100 top HVGs did not contain FOXM1. Furthermore, even if FOXM1 was added into the set of HVGs, we found that the existing correlational network methods could not prioritize FOXM1 as a key gene based on various centrality measurements, indicating the importance of proper gene selection by accounting for the latent-temporal dynamics of gene expression.

***Incorporating prior network topology into PROB***

The prior information of networks, such as gene co-expressions or protein-protein interactions, could be utilized to construct an initial network prior to applying PROB. For the example of breast cancer dataset, since the network size is large (100 genes as selected above), to improve the precision of network inference, we incorporated high level of co-expressions into PROB to narrow down the number of variables during Bayesian inference. We calculated mutual information for each pair of selected genes. By assuming sparse network structure, the top 5% edges were retained as initial (undirected) network. The adjacent matrix of the initial MI network was denoted as $MI_{n\times n}$. In Equation (15), the matrix $X^{(i)}$ was modified according to $MI_{n\times n}$, to form a new matrix $X_{MI}^{(i)}$. More specifically, if $MI_{i,j}$=0, then the *j*-th row of $X^{(i)}$ was removed to reduce the number of predictors in the Bayesian-Lasso regression.

***Eigenvector centrality***

We identified the hub gene in the GRN based on an eigenvector centrality measure according to singular value decomposition method [3]. Denote the mean of the posterior distributions of $a_{ij}$ as $m_{ij}$, and $M=\left(m_{ij}\right)_{n\times n}$. We subject *M* to singular value decomposition. We calculated the principal eigenvector of *MM^T^* and denoted it *H=*(*h_1_*, *h_2_*, …, *h_n_*). The hub score of node *i* was defined as *h_i_*. The gene with greatest hub score was identified as a hub gene for further analysis and validation.

**Supplementary references**

1. Guo CC, Majewski T, Zhang L, Yao H, Bondaruk J, Wang Y, et al. Dysregulation of EMT Drives the Progression to Clinically Aggressive Sarcomatoid Bladder Cancer. Cell reports. 2019;27(6):1781-93.e4. Epub 2019/05/09. doi: 10.1016/j.celrep.2019.04.048. PubMed PMID: 31067463; PubMed Central PMCID: PMCPMC6546434.

2. Desmedt C, Piette F, Loi S, Wang Y, Lallemand F, Haibe-Kains B, et al. Strong time dependence of the 76-gene prognostic signature for node-negative breast cancer patients in the TRANSBIG multicenter independent validation series. Clinical cancer research : an official journal of the American Association for Cancer Research. 2007;13(11):3207-14. Epub 2007/06/05. doi: 10.1158/1078-0432.Ccr-06-2765. PubMed PMID: 17545524.

3. Kleinberg JM. Authoritative sources in a hyperlinked environment. Journal of the ACM. 1999;46(5):604-32. doi: 10.1145/324133.324140.
