# Supplementary material for: Inferring latent temporal progression and regulatory networks from cross-sectional transcriptomic data of cancer samples
Source: PLoS Comput Biol. 2021 Mar 5;17(3):e1008379. doi: 10.1371/journal.pcbi.1008379 (PMC7968745; doi:10.1371/journal.pcbi.1008379)
Supplement: S6 Text — (DOCX) [file pcbi.1008379.s022.docx]

**Text S6. Clinical relevance of FOXM1 to breast cancer**

We found that FOXM1 belonged to an ascending group (**Fig 6a**), indicating its association with cancer progression. We thereby verified the clinical relevance of FOXM1 by examining the prognostic roles of FOXM1 in breast cancer patients based on GSE7390 dataset (*n*=196) [1]. A risk score was defined for each patient based on the expression level of FOXM1 according to COX PH model. High expression level of FOXM1 corresponds to high risk, and low expression level of FOXM1 corresponds to low risk. In this way, patients were divided into two groups: high-risk group and low-risk group. The optimal cutoff value of the risk score was determined using the ROC method [2]. **Fig 6d-f** show the K-M survival curves of patients in high FOXM1 expression group (blue) and low FOXM1 expression group (red) with respect to distant-metastasis-free survival (DMFS; **Fig 6d**), relapse-free survival (RFS; **Fig 6e**) and overall survival (OS; **Fig 6f**), respectively. All these results demonstrated that up-regulation of FOXM1 was significantly associated with the poor prognosis of breast cancer patients, as assessed by log-rank test *p* values. Furthermore, we performed permutation test of the prognostic power of FOXM1 using a bootstrapping approach. We randomly selected 10000 genes from the whole transcriptome and evaluated the prognostic association of each gene with the RFS using Wald test p value. Among the 10000 “random gene” p values, only 1.46% were less than the p value of FOXM1 (i.e., 0.0018). Therefore, the p value from the permutation test was 0.0146, indicating non-randomness of FOXM1 and verifying the statistical significance of the prognostic power of FOXM1.

Moreover, we found that FOXM1 expression was associated with the therapeutic responses of breast cancer patients (**Fig S6**). Breast cancer patients who received endocrine therapy (**Fig S6a**) or chemotherapy (**Fig S6b**) were included into the K-M survival analysis. Log-rank test p-value was used to assess the prognostic significance.

**Supplementary references**

1. Desmedt C, Piette F, Loi S, Wang Y, Lallemand F, Haibe-Kains B, et al. Strong time dependence of the 76-gene prognostic signature for node-negative breast cancer patients in the TRANSBIG multicenter independent validation series. Clinical cancer research : an official journal of the American Association for Cancer Research. 2007;13(11):3207-14. Epub 2007/06/05. doi: 10.1158/1078-0432.Ccr-06-2765. PubMed PMID: 17545524.

2. Xiaoqiang Sun XL, Mengxue Xia, Yongzhao Shao, Xiaohua Douglas Zhang. Multicellular gene network analysis identifies a macrophage-related gene signature predictive of therapeutic response and prognosis of gliomas. Journal of Translational Medicine. 2019;17:159. doi: 0.1186/s12967-019-1908-1.
