# Supplementary material for: Inferring latent temporal progression and regulatory networks from cross-sectional transcriptomic data of cancer samples
Source: PLoS Comput Biol. 2021 Mar 5;17(3):e1008379. doi: 10.1371/journal.pcbi.1008379 (PMC7968745; doi:10.1371/journal.pcbi.1008379)
Supplement: S7 Text — (DOCX) [file pcbi.1008379.s023.docx]

**Text S7. Validation of predicted FOXM1-targets interactions**

***Validation using gene expression data***

To validate the expression changes of the predicted targeted genes (including ASPM, CDCA8, KIF2C, MCM10, MELK, NCAPG, SHCBP1 and STIL (**Fig 6a**)) following FOXM1 perturbation, we analyzed microarray gene expression data in MCF-7 cells that were treated with DMSO (control) or Thiostrepton (FOXM1 inhibitor) for 6 hours (GSE40762 [1]). The differential expression of the above 8 genes between control condition and FOXM1 inhibition condition was examined to test whether they were down-regulated after FOXM1 inhibition. The statistical significance was assessed using Wilcoxon rank sum test (one-tailed) p values. **Fig 6b** shows that except for SCCBP1 and STIL, other 6 genes were significantly down-regulated after FOXM1 inhibition.

***Validation using ChIP-seq data***

To verify whether FOXM1 binds to some of the predicted targeted genes, we used ChIP-seq data in both MCF-7 cell line (ER+) and MDA-MB-231 cell line (ER-) (GSE40762 [1]) to analyze binding of FOXM1.

The ChIP-seq analysis for peak calling was performed using a standard procedure as follows: (1) All the downloaded raw data were subjected to Trim Galore (<http://www.bioinformatics.babraham.ac.uk/projects/trim_galore/>) for filtering low-quality sequence reads and trimming the 3’/5’ adapters. (2) The cleaned data was aligned to the Homo sapiens reference genome (assembly hg19, Genome Reference Consortium GRCh37, Feb. 2009) using bowtie2 V2.3.5 [2] with default setting. (3) Each aligned sample in the treatment group was subjected to MACS2 V 2.1.2 [3] software to identify the read-enriched regions (i.e. biding sites) in comparison with the input sample in the control group from the same cell lines as Chip libraries, respectively. Differential enriched peaks (FDR < 0.05) were identified through MCAS2 to account for local enrichment biases. (4) For more suitable visualization, the alignment results were converted to bigwig format using a series of softwares, e.g. MACS2 bdgcmp, bedtools slop (V 2.29.0 [15]) and UCSC toolkits including bedClip V 332 and bedgraphtobigwig V 323. (5) The peaks and alignment results were visualized through IGV V2.6.3 [4] software to compare the peaks and aligned reads on the predicted targeted genes of FOXM1.

The results on both MCF-7 cell line (ER+) (**Fig** **6c-e**) and MDA-MB-231 cell line (ER-) (**Fig** **6f-h**) showed that FOXM1 binds ASPM, CDCA8 and KIF2C. In addition, we analyzed another set of ChIP-seq data in the human mammary epithelial cells (HMEC) (GSE62425) [5]. It was found that FOXM1 did bind ASPM and KIF2C but not CDCA8. Comparison of ChIP-seq analysis results on HMEC and MCF-7 or MDA-MB-231 cell lines might indicate oncogenic role of the emerging binding of FOXM1 to certain targeted genes during breast tumor formation.

In addition, we confirmed the predicted regulation of FOXM1 on ASPM, CDCA8 and KIF2C using both microarray data and RNA-seq data in two breast cancer cell lines. A set of microarray data (GSE2222) [6] in BT-20 breast cancer cells treated with mock transfection or GFP siRNA (control) or FOXM1 siRNA was employed to analyze the expression levels of the above three genes (**Fig S9a-c**). Using RNA-seq data of MCF-7 breast cancer cells (GSE58626) [7], we analyzed the differential expressions of the above three genes after FOXM1 inhibition by using small molecule compound FDI-6 (NCGC00099374) that specifically inhibits FOXM1 (**Fig S9d-f**). The statistical significance was assessed using Wilcoxon rank sum test (one-tailed) p values. The knockdown or silence of FOXM1 significantly reduced the expressions of the above three genes.

**Supplementary references**

1. Sanders DA, Ross-Innes CS, Beraldi D, Carroll JS, Balasubramanian S. Genome-wide mapping of FOXM1 binding reveals co-binding with estrogen receptor alpha in breast cancer cells. Genome biology. 2013;14(1):R6. Epub 2013/01/26. doi: 10.1186/gb-2013-14-1-r6. PubMed PMID: 23347430; PubMed Central PMCID: PMCPMC3663086.

2. Langmead B, Salzberg SL. Fast gapped-read alignment with Bowtie 2. Nat Methods. 2012;9(4):357-9. Epub 2012/03/06. doi: 10.1038/nmeth.1923. PubMed PMID: 22388286; PubMed Central PMCID: PMCPMC3322381.

3. Zhang Y, Liu T, Meyer CA, Eeckhoute J, Johnson DS, Bernstein BE, et al. Model-based analysis of ChIP-Seq (MACS). Genome Biol. 2008;9(9):R137. doi: 10.1186/gb-2008-9-9-r137. PubMed PMID: 18798982; PubMed Central PMCID: PMCPMC2592715.

4. Thorvaldsdottir H, Robinson JT, Mesirov JP. Integrative Genomics Viewer (IGV): high-performance genomics data visualization and exploration. Brief Bioinform. 2013;14(2):178-92. doi: 10.1093/bib/bbs017. PubMed PMID: 22517427; PubMed Central PMCID: PMCPMC3603213.

5. Lanigan F, Brien GL, Fan Y, Madden SF, Jerman E, Maratha A, et al. Delineating transcriptional networks of prognostic gene signatures refines treatment recommendations for lymph node-negative breast cancer patients. The FEBS journal. 2015;282(18):3455-73. Epub 2015/06/23. doi: 10.1111/febs.13354. PubMed PMID: 26094870.

6. Wonsey DR, Follettie MT. Loss of the Forkhead Transcription Factor FoxM1 Causes Centrosome Amplification and Mitotic Catastrophe. Cancer Research. 2005;65(12):5181-9. doi: 10.1158/0008-5472.CAN-04-4059.

7. Gormally MV, Dexheimer TS, Marsico G, Sanders DA, Lowe C, Matak-Vinković D, et al. Suppression of the FOXM1 transcriptional programme via novel small molecule inhibition. Nature Communications. 2014;5(1):5165. doi: 10.1038/ncomms6165.
