# Supplementary material for: Inferring latent temporal progression and regulatory networks from cross-sectional transcriptomic data of cancer samples
Source: PLoS Comput Biol. 2021 Mar 5;17(3):e1008379. doi: 10.1371/journal.pcbi.1008379 (PMC7968745; doi:10.1371/journal.pcbi.1008379)
Supplement: S8 Text — (DOCX) [file pcbi.1008379.s024.docx]

**Text S8. GRN kinetic signature**

We hypothesized that the kinetic features of the core module or subnetwork of the reconstructed GRN could be used as a biomarker predictive of cancer progression. We tested whether the kinetic rates of FOXM1-targeted genes were associated with prognosis in breast cancer. The kinetic rate of each target gene ($X_{i}$) contributed by FOXM1 can be expressed as $R_{i}=a_{i,FOXM1}X_{i}\cdot FOXM1$ $\left( i=1, 2, \cdots, 8 \right)$based on the dynamic model of the GRN (Equation (10)). Therefore, we defined a new vector $R=(R_{1}, R_{2}, \ldots, R_{8}$) representing the kinetic features of FOXM1 subnetwork for each patient, called a GRN kinetic signature, to predict relapse in breast cancer patients. A Cox model [1] was used to evaluate the prognostic roles of the GRN kinetic signature in breast cancer using multiple independent datasets (GSE2990 [2], GSE12093 [3] and GSE5327 [4]).

We employed a bootstrapping approach to test whether the randomly-selected genes as targets of FOXM1 possessed stronger association significance than our predicted FOXM1-targets interactions. More specifically, each time, 8 genes were randomly selected from the whole transcriptome to construct presumptive interactions between FOXM1, referred to FOXM1-random targets. According to the law of mass action, the kinetic rate of each target gene expression ($Y_{i}$) contributed by FOXM1 is proportional to $\tilde{R}_{i}=Y_{i}\cdot FOXM1$ $\left( i=1,2,\cdots,8 \right).$ We evaluated the significance of prognostic association of FOXM1-random targets ($\tilde{R}_{1},\tilde{R}_{2},\cdots, \tilde{R}_{8}$) with the RFS using Wald test p value based on COX model [1]. The above process was repeated 10000 times. The estimated distribution of Wald test p values of FOXM1-random targets was compared to the Wald test p value of FOXM1-predicted targets. The probability that Wald test p values of FOXM1-random targets were less than the Wald test p value of FOXM1-predicted targets was calculated, resulting in a permutation test p value. We employed three independent datasets (GSE2990 [2], GSE12093 [3] and GSE5327 [4]) to perform the above significance test.

**Supplementary references**

1. Cox DRJJotRSS. Regression models and life-tables. 1972;34(2):187-220.

2. Christos S, Pratyaksha W, Sherene L, Adrian H, Steve F, Johanna S, et al. Gene expression profiling in breast cancer: understanding the molecular basis of histologic grade to improve prognosis. J Natl Cancer Inst. 2006;98(4):262-72.

3. Zhang Y, Sieuwerts AM, McGreevy M, Casey G, Cufer T, Paradiso A, et al. The 76-gene signature defines high-risk patients that benefit from adjuvant tamoxifen therapy. Breast Cancer Research. 2009;116(2):303-9. doi: 10.1007/s10549-008-0183-2.

4. Minn AJ, Gupta GP, Padua D, Bos P, Nguyen DX, Nuyten D, et al. Lung metastasis genes couple breast tumor size and metastatic spread. Proceedings of the National Academy of Sciences of the United States of America. 2007;104(16):6740-5. Epub 2007/04/11. doi: 10.1073/pnas.0701138104. PubMed PMID: 17420468; PubMed Central PMCID: PMCPMC1871856.
