# Supplementary material for: Inferring latent temporal progression and regulatory networks from cross-sectional transcriptomic data of cancer samples
Source: PLoS Comput Biol. 2021 Mar 5;17(3):e1008379. doi: 10.1371/journal.pcbi.1008379 (PMC7968745; doi:10.1371/journal.pcbi.1008379)
Supplement: Attachment — Submitted filename: Response to Reviewers.pdf [file pcbi.1008379.s026.pdf]

Response to Reviewer #1

Gene regulatory network (GRN) reconstruction methods usually require time-course gene expression data or perturbation experiment data. However, most available omics data from cross-section studies of cancer patients often lack temporal information, making it challenging to reconstruct the GRN for the studied cancer progression. In this work, Sun et al. present a latent-temporal progression-based bayesian method, PROB, for the inference of GRNs from the cross-sectional transcriptomic data of tumor samples. Compared with other GRN methods, the proposed method has a number of advantages: (1) First, it was designed specifically to reconstruct GRNs from tumor samples, which utilizes a lot of cancer-specific information (staging) that are not utilized by others general methods (such as GENIE3). (2) Second, the authors mathematically proved the robustness of the proposed methods. The robustness is especially important in reconstructing GRNs from cancer samples, which are usually highly noisy. Third, the proposed method requires minimal prior knowledge. For example, in other GRN reference methods, the root (node) is usually needed to pick out by the users based on their prior knowledge. The proposed methods can automatically learn the root using a proposed distance-based approach. This significantly improves the usability of the proposed method. Overall, I think that this is a fantastic method and well fit the authorship of the journal. I highly recommend the publication of this manuscript.

R: Thank you very much for your positive comments on our manuscript.

I only have a few minor comments:

(1) In equation (5), the authors described that the root is determined as the patient (x) with the largest distance to the patients the maximal grade score (e.g., stage 4). I would suggest limiting the candidate x \ patients with the smallest grade (e.g., grade 0). If not, theoretically, it is likely that x0 will come from a later stage (e.g., stage 2 or even stage 4 (e.g., because of a few outliers that produce a considerable distance). In that case, the root x0 will come from stage 2 or stage 4, which would not make much sense to me. As the stage information is available, I would suggest to narrow down the selection of x0 among patients in stage 0

R: Thank you for your good suggestion. We agree with your comment on the potential influence of a few outliers. According to your advice, we have revised it as follows (now Equation (9) in the revised manuscript):

$$x_0 = \arg\max_{x \in \{x_{\min}\}} TPD(x, x_{ref})$$

"where x_{ref} is a randomly selected patient from the maximal grade subpopulation. The selection of x_0 was limited among patients with the smallest grade (i.e., $\{x_{min}\}$) to eliminate potential influence of a few outliers in the data."

We also modified the code correspondingly and tested the influence of this modification on the two real datasets used in this study and detected no difference at least for the studied two cases.

(2) In this work, the authors simulate Gaussian noise to examine the robustness of the method. I am amazed by the robustness of the method to Gaussian noise. However, the Gaussian noise is usually well dealt with. Did the authors also simulate other types of noise (e.g., drop-out noise is also a very common noise type)? It would be great if the methods can also be tested against other noises.

R: Thank you for your insightful comments and good suggestion.

In addition to the above Gaussian noises, we also tested the robustness of our method against **exponential noises** (Figure S5). The gene expressions were randomly perturbed by using multiplicative exponential noises generated from the exponential distribution with mean ranging from 0 to 0.3.

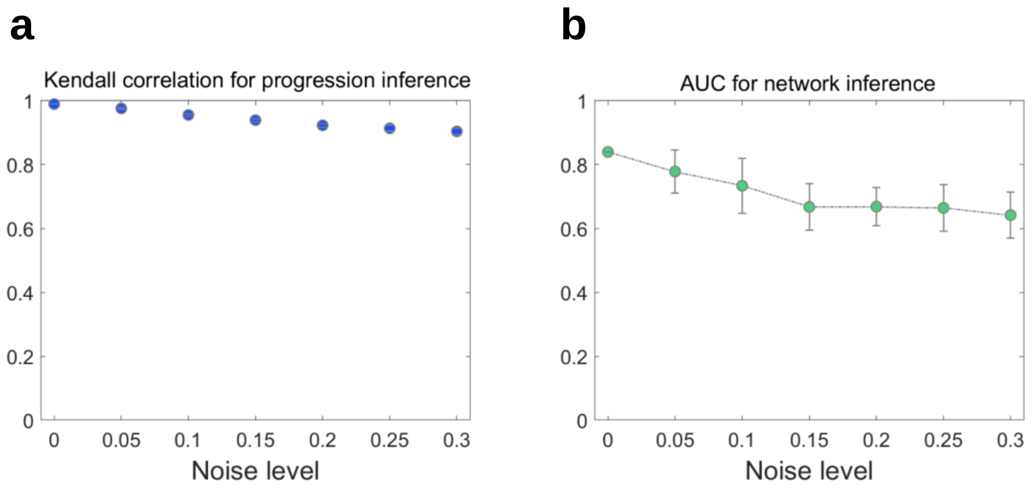

Figure S5. Testing the robustness of PROB against exponential noises. The noises were generated from the exponential distribution with mean ranging from 0 to 0.3. (**a**) Kendall correlation for evaluating the accuracy of the temporal progression inference. (**b**) AUC for evaluating the robustness of the GRN inference.

We agree that the drop-out noise is also a common noise type, particularly for single-cell RNA-sequencing data, but may not be the major source of noise in RNA-seq data of cancer samples. Since our study is intended primarily for the transcriptomic data of cancer samples (e.g., microarray and RNA-seq data) whose typical noise may come from sample purity, so we used Gaussian noise and exponential noises (always positive) to imitate this situation. We acknowledge that the drop-out noise is indeed more difficult to dealt with. Nevertheless, we have figured out a way to deal with the drop-out noise under a unified framework of our method, which will be investigated in our future study oriented specifically to scRNA-seq data.

Response to Reviewer #2

General Comments

Authors developed a latent-temporal progression-based Bayesian method, PROB, for inferring gene regulatory networks (GRNs) from the cross-sectional transcriptomic data of tumor samples. Mathematical proofs and numerical verification are provided to support the robustness of PROB to the measurement variabilities in the data. Benchmarking PROB with alternative methods of GRN inference shows advantages of PROB in both pseudotime inference and GRN inference. Authors also demonstrated the applications of PROB for identification of key regulators of cancer progression or drug targets as well as performed validations experiments. Overall, the manuscript is clear and accessible.

R: Thank you for your positive feedback. We now have revised the manuscript according to your comments and suggestions.

Specific comments

Pages 9-12: Section "Latent-temporal progression-based Bayesian (PROB) method to infer GRN"

This section can be rewritten more compactly to improve reading experience. Either put all details in the main text or keep only the main idea in the main text and leave the details in the supplementary text. Current main text is an abridged version of Text S1 and does not read consistent.

R: Thanks for your good suggestion.

To keep text consistent and symbols in Algorithm 1 clearly defined, we decided to put all details of the method into the main text, except for the details of assumption and derivation of the dynamical modeling, which are now provided in Text S1 ("Progression-dependent dynamic modeling of the GRN").

Page 9: "… the root was automatically identified with the aid of staging information."

What is "root" meant here and in the subsequent context? Does it adapt a generally used and accepted mathematical meaning? Should it be called "optimizer"?

R: The "root" here means the starting point in the latent temporal progression trajectory. It is widely used and accepted for pseudo-temporal trajectory inference in the previous papers of single-cell RNA-seq data analysis, for instance:

Farrell JA, Wang Y, Riesenfeld SJ, Shekhar K, Regev A, Schier AF. Single-cell reconstruction of developmental trajectories during zebrafish embryogenesis. Science. 2018;360(6392):eaar3131. (PMID: 29700225)

Sun H, Wen X, Li H, et al. Single-cell RNA-seq analysis identifies meniscus progenitors and reveals the progression of meniscus degeneration. Annals of the Rheumatic Diseases 2020;79:408-417. (PMID: 31871141)

To make it clearer, we described it as “root of the progression trajectory”. The above sentence has been revised as follows:

“… the root of the progression trajectory was automatically identified with the aid of staging information”

Page 11: “The above model is then transformed into a linear regression model …”
I think it’s better phrased as “a linear system”. Rigorously speaking Y is not linear in X.

R: Sorry for confusion due to missing information in the original main text. Now we have included details into the main text and the final model reads (Equation (15)):

$$Y_i = A_i X^{(i)} + \varepsilon_i \ , \quad (i = 1, 2, \cdots, n),$$

We can see that Y_i is linear in $X^{(i)}$. So we decided to keep “linear regression model” in the revised manuscript, provided that no confusion would occur now.

Page 12: “Therefore, the above theorem theoretically guarantees the consistency and robustness of the estimates of the regulatory coefficients.”
It can be elaborated more the role of Theorem 1; for example, add 2-3 lines of equations.

R: Thanks for your suggestion. We have added the following corollary and implications of Theorem 1.

“A corollary of the above theorem is that the mapping $s \mapsto [a_{ij}(s)]_{n\times n}$ defined by Equation (10) is continuous under certain appropriate metric. More specifically, for two trajectories s and $\tilde{s}$, if the difference between the two inferred regulatory coefficients $[a_{ij}(s)]_{n\times n}$ and $[a_{ij}(\tilde{s})]_{n\times n}$ is significantly larger than 0, then the difference between s and $\tilde{s}$ should not be arbitrarily small. This implies that if the inferred regulatory networks for two progressions are largely different, then the two progressions should have different trajectories and thus distinct clinical outcomes. Hence, Theorem 1 also suggests that GRN-based signatures may be used for predicting or controlling cancer progression.”

Page 12: pseudo-code of PROB
Variable E is not defined in the main text.
Definition of phi should be readdressed in the algorithm.
Variable/function PPD is not defined.

R: Thanks for pointing them out.

Variable E has now been defined in the Methods section of the main text.

Definition of ψ_0 has been included in the algorithm.

PPD was a typo and has been corrected to TPD.

Page 13: "To this end, we defined an outgoing causality score (OCS) …"
Does OCS have any mathematical/statistical meaning?

R: The OCS represents the sum of regulatory strengths from gene i to its target genes ($\sum_{j=1}^{n} m_{ji} X_j^k X_i^k$). It was defined in view of the Equation (10) (dynamic model of gene regulation) based on the mass action kinetics. The OCS quantifies the outgoing regulatory potential of a give gene and takes large value for regulators but small values for targets. Intuitively, for a set of genes with known regulatory relationship (regulators and targets), if gene i is a regulator, then the OCS value should be large since many values of m_{ji} (absolute value of mean of a_{ji}) should be greater than 0; If gene i is a target, then the OCS value should be small since most values of m_{ji} should be zero or small enough.

We have added the following text into the main text:

"The OCS is defined in accordance of the Equation (10) based on the mass action kinetics and quantifies the outgoing regulatory potential of a give gene."

Page 21: "The code for PROB is available at https://github.com/SunXQlab/PROB."
This page does not exist.

R: We are sure that the code has been uploaded into Github with URL: https://github.com/SunXQlab/PROB

We have confirmed that the above URL is valid, which has also been tested by other colleagues. If the problem still exists, please try another browser. Thanks.

Page 28: Figure 2d
Why does not AUC decrease monotonically with CV?

R: Figure 2 illustrates the result of one realization of the simulation under specific noise level. Due to random variations, the AUC in Figure 2d does not monotonically decrease. The multiple simulation results demonstrated that the mean value of AUC decreases monotonically with CV, which were provided in supplementary **Figure S4C**.

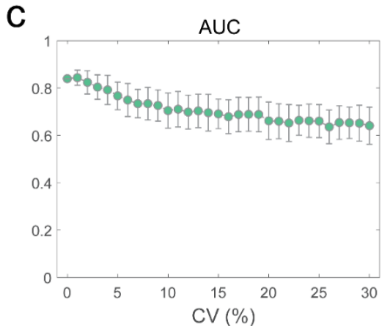

(Figure S4C)

Page 32: Figure 6: Details related to survival analysis are missing (e.g. how authors define high and low risk groups. What is their cut-off?) Also role of FOXM1 on breast cancer progression is known and citations are missing.

R: Thanks for you pointing this out. We now have added the details of survival analysis in Figure 6 into the Text S6.

"A risk score was defined for each patient based on the expression level of FOXM1 according to COX PH model. High expression level of FOXM1 corresponds to high risk, and low expression level of FOXM1 corresponds to low risk. In this way, patients were divided into two groups: high-risk group and low-risk group. The optimal cutoff value of the risk score was determined using the ROC method [2]."

The citations for the role of FOXM1 in breast cancer have been added (Ref. [46]).

Page S10: "The codes are available at https://github.com/dongbusun/PROB."
This page does not exist.

R: The above URL has been updated to the following one:

https://github.com/SunXQlab/PROB

We confirmed that this URL is valid, which has been tested and confirmed by other colleagues.

If the problem still exists, please try another browser. Thanks.

Page S15: "PROB applied to realistic datasets"
Realistic -> real

R: Thanks for pointing it out. We have corrected it.
